# Efficacy and safety of selegiline for the treatment of Parkinson's disease: A systematic review and meta-analysis

**DOI:** 10.3389/fnagi.2023.1134472

**Published:** 2023-04-11

**Authors:** Ke Wang, Ze-Hui Liu, Xin-Ya Li, Yan-Fei Li, Jia-Rui Li, Jiao-Jiao Hui, Jing-Xuan Li, Jun-Wen Zhou, Zhan-Miao Yi

**Affiliations:** ^1^Department of Pharmacy, Peking University Third Hospital, Beijing, China; ^2^Department of Pharmacy, Xuanwu Hospital of Capital Medical University, Beijing, China; ^3^Department of Pharmacy, Aerospace Central Hospital, Beijing, China; ^4^Department of Pharmacy, The First People's Hospital of Xianyang, Shaanxi, China; ^5^Department of Pharmacy, The Second Hospital of Hebei Medical University, Hebei, China; ^6^Health Economics Research Centre, Nuffield Department of Population Health, University of Oxford, Oxford, United Kingdom; ^7^Institute for Drug Evaluation, Peking University Health Science Center, Beijing, China

**Keywords:** Parkinson's disease, Unified Parkinson's Disease Rating Scale, Hamilton Depression Rating Scale, Webster Rating Scale, selegiline, adverse events

## Abstract

**Background:**

Drug efficacy generally varies with different durations. There is no systematic review analyzing the effect of selegiline for Parkinson's disease (PD) on different treatment duration. This study aims to analyze how the efficacy and safety of selegiline changes for PD over time.

**Methods:**

PubMed, the Cochrane Library, Embase, China National Knowledge Infrastructure and Wanfang Database were systematically retrieved for randomized controlled trials (RCTs) and observational studies of selegiline for PD. The search period was from inception to January 18th, 2022. The efficacy outcomes were measured by the mean change from baseline in the total and sub Unified Parkinson's Disease Rating Scale (UPDRS), Hamilton Depression Rating Scale (HAMD) and Webster Rating Scale (WRS) scores. The safety outcomes were measured by the proportion of participants having any adverse events overall and that in different system organ classes.

**Results:**

Among the 3,786 studies obtained, 27 RCTs and 11 observational studies met the inclusion criteria. Twenty-three studies reported an outcome which was also reported in at least one other study, and were included in meta-analyses. Compared with placebo, selegiline was found with a stronger reduction of total UPDRS score with increasing treatment duration [mean difference and 95% CIs in 1 month: −3.56 (−6.67, −0.45); 3 months: −3.32 (−3.75, −2.89); 6 months: −7.46 (−12.60, −2.32); 12 months: −5.07 (−6.74, −3.41); 48 months: −8.78 (−13.75, −3.80); 60 months: −11.06 (−16.19, −5.94)]. A similar trend was also found from the point estimates in UPDRS I, II, III, HAMD and WRS score. The results of observational studies on efficacy were not entirely consistent. As for safety, compared with placebo, selegiline had higher risk of incurring any adverse events [rate: 54.7% vs. 62.1%; odd ratio and 95% CIs: 1.58 (1.02, 2.44)], with the excess adverse events mainly manifested as neuropsychiatric disorders [26.7% vs. 31.6%; 1.36 (1.06, 1.75)] and no significant change over time. The statistically difference in overall adverse event between selegiline and active controls was not found.

**Conclusion:**

Selegiline was effective in improving total UPDRS score with increasing treatment duration, and had a higher risk of incurring adverse events, especially the adverse events in the neuropsychiatric system.

**Systematic review registration:**

https://www.crd.york.ac.uk/prospero/, identifier: PROSPERO CRD42021233145.

## Introduction

Parkinson's disease (PD) is an illness characterized by the loss of dopaminergic neurons in the substantia nigra. Its typical clinical manifestation includes bradykinesia, rigidity, rest tremor and disturbances in balance (Obeso et al., 2017). The prevalence of PD is increasing over years, with the global prevalence increasing from 2.5 million in 1990 to 6.1 million in 2016, which brings heavy burdens to the society (GBD 2016 Neurology Collaborators, 2018; Simon et al., 2020). Currently, pharmacological therapy is the main treatment for PD (Armstrong and Okun, 2020). Monoamine oxidase type B (MAO-B) inhibitors are one of the medications commonly used for PD treatment (NICE, 2017; Grimes et al., 2019; Tan et al., 2022).

Selegiline was the only MAO-B inhibitor in the past few decades (Magyar, 2011). It is an irreversible and selective MAO-B inhibitor which blocks dopamine metabolism and inhibits dopamine degradation, thus increasing dopamine and improving motor symptoms of patients (Moore and Saadabadi, 2022). Meanwhile, selegiline blocks synaptic dopamine reuptake and prolongs the duration of dopamine action, in this way it can help improve the function of dopaminergic neurons (Nagatsu and Sawada, 2006). In addition, selegiline can enhance the effect of improving akinesia and mitigate levodopa-induced dyskinesia when it is used with levodopa (Tábi et al., 2020).

Unified Parkinson's Disease Rating Scale (UPDRS) was the most widely used tool to gauge the severity and progression of PD in patients. The UPDRS Version 3.0 has four components covering mentation, behavior, and mood (UPDRS I), activities of daily living (UPDRS II), motor symptoms (UPDRS III) and complications of therapy (UPDRS IV) (Fahn et al., 1987). The UPDRS demonstrates high internal consistency and inter-rater reliability, shows moderate construct validity, and has a stable factor structure (Ramaker et al., 2002). Hamilton Depression Rating Scale (HAMD), which has adequate reliability and high validity, was recommended to be used for depression screening in PD (Hamilton, 1960; Miyasaki et al., 2006; Chai and Ho, 2021). Webster Rating Scale (WRS), with a few studies showed its moderate reliability, can also indicate the severity of PD and the clinical impairment (Webster, 1968; Ginanneschi et al., 1988).

Selegiline was found with increasing improvement of UPDRS, HAMD and WRS scores over time (Pålhagen et al., 2006; Mizuno et al., 2017). However, there is no systematic review that summarizes and analyses the literatures on such trend at present. Previous systematic reviews of selegiline merely focus on the overall efficacy and safety of selegiline monotherapy or combination therapy. Some of them had mixed results. For example, Ives et al. (2004) found UPDRS scores were improved with selegiline when compared with placebo for UPDRS II and UPDRS III. However, Su et al. (2014) reported that no significant improvement was found. Finally, none of them included observational studies, though observational data can serve as convincing and valuable evidence (van den Heuvel et al., 2021).

The aim of our study is to perform a systematic review and meta-analysis of RCTs and observational studies, to assess the efficacy and safety of selegiline for the treatment of PD on different treatment durations. In addition, the impacts of selegiline on the incidence of adverse events in various systems will also be explored in detail in our study.

## Materials and methods

### Search strategy

We performed our study by searching for studies on selegiline for PD in the following databases from inception of each database to January 18th, 2022: PubMed (from 1996), the Cochrane Library (2021 issue 12) Embase (from 1980), China National Knowledge Infrastructure (from 1999) and Wanfang Database (from 2001). We also limited the language of literature to English and Chinese. We chose the keywords “selegiline” and “Parkinson” as search terms. The Boolean logic “AND” was used to connect the two terms. The protocol of this meta-analysis and systematic review was registered in PROSPERO (No. CRD 42021233145).

### The selection of study and outcome measures

After the screening of the title and abstract, researchers inspected all studies by examining the full articles. Three independent authors (ZHL, JRL, and YFL) manually screen the records of eligible studies through title, abstract and full text, and disagreements were resolved *via* discussion. The inclusion criteria were specified as following: (1) RCTs or observational studies; (2) patients diagnosed with PD; (3) patients received selegiline monotherapy or selegiline combined with other treatment; (4) outcomes: change from baseline in UPDRS score including total UPDRS score, UPDRS I, UPDRS II, and UPDRS III, HAMD score, WRS score, proportion of patients having any adverse events overall and that in different system organ classes.

### Data collection and research quality evaluation

According to the predesigned data acquisition form, data extraction was performed by different independent investigators (KW, XYL). The extracted information includes the authors' participant characteristics, publication year, countries, dosages, treatment durations and outcomes. The two investigators independently evaluated the methodological quality of included studies. Risk bias of included RCTs was evaluated with Cochrane risk of bias assessment tools (Higgins et al., 2011). The methodological quality of included observational studies was evaluated with the Newcastle–Ottawa scale (NOS) (Wells et al., 2014). When relevant data were missing in the included studies, we contacted their authors for clarification. All disagreements on data collection, abstraction and quality assessment were resolved by investigator consensus agreement.

### Statistical analysis

In this review, we described studies that covered outcome results at relevant evaluation date. We performed the meta-analyses for the efficacy outcomes at each available follow-up periods for the outcome assessment separately, and for the safety outcomes for each type of treatment of the control arm separately. Those periods should be reported by at least two studies. Statistical analysis was carried out using Revman Manager 5.3 software (Cochrane Collaboration, Oxford, UK). Mean difference (MD) and 95% confidence interval (95% CI) were estimated for continuous data (changes from baseline in UPDRS score, HAMD score or WRS score), and dichotomous data (incidence of adverse events) were expressed as odds ratio (OR) and 95% CI. Cochrane *Q*-statistic and *I*^2^-test were adopted to test the heterogeneity of the selected studies. If the heterogeneity was small (*P* ≥ 0.1, *I*^2^ < 50%), the combined effect size was calculated by adopting the fixed effect model (Higgins and Thompson, 2002). Otherwise, the random effect model was adopted. Sensitivity analysis was performed to test the robustness of the results by excluding studies with distinct outcome differences. Finally, publication bias was examined by funnel plot with 10 or more included studies. All tests were two-sided and a value of *P* < 0.05 was regarded as statistically significant.

## Results

### Study inclusion

**Figure 1:**
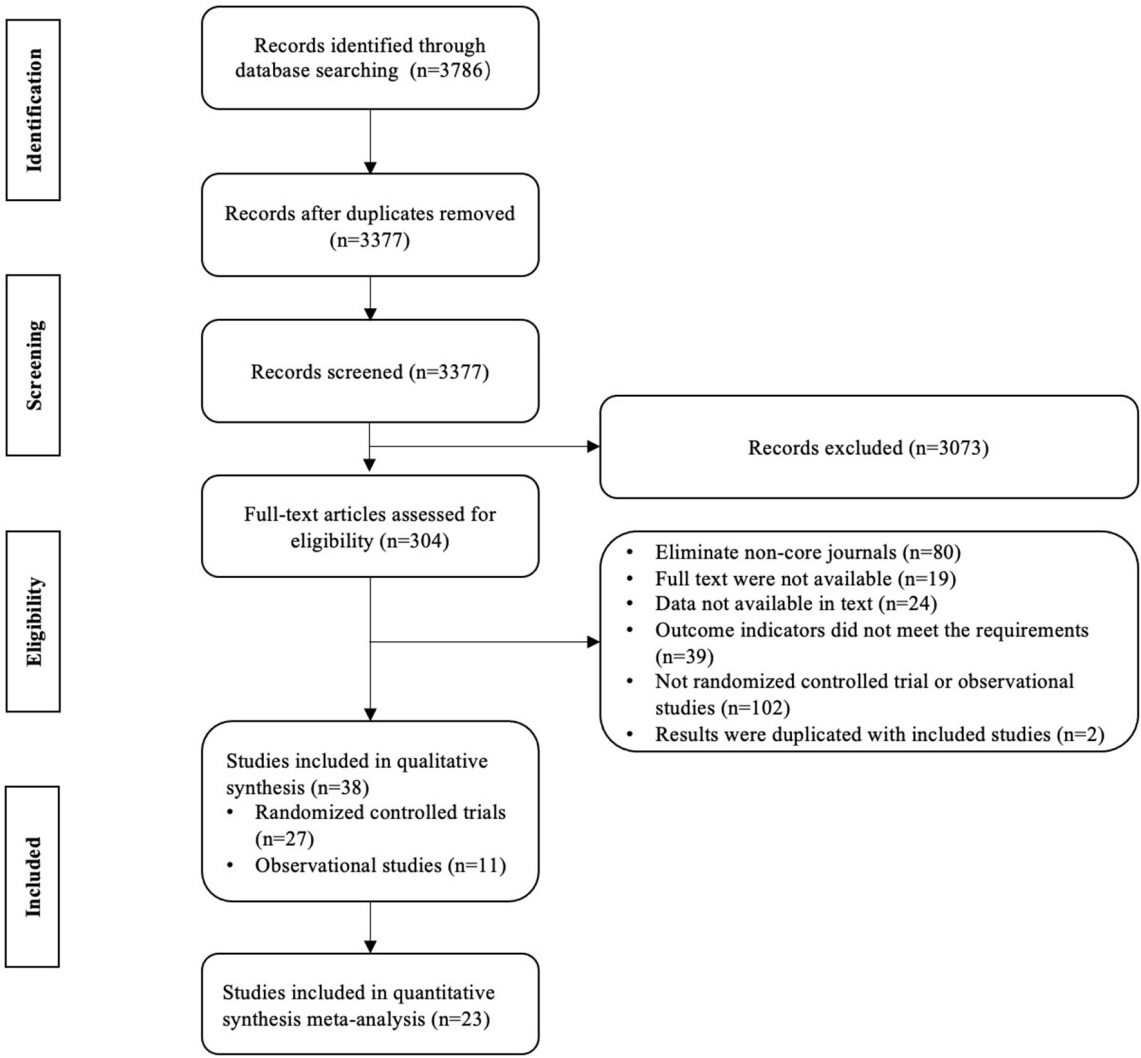
PRISMA flowchart of literature retrieval and selection.

A total of 3,786 studies were obtained initially and 409 duplicate studies were removed. After the title/abstract screening, 3,073 studies were excluded, and 304 studies were qualified for the full text screening. We excluded 266 studies for the following reasons: 102 studies were not RCTs or observation studies, 80 with Chinese language were from non-core journals, 19 full texts were not available, 24 data were not available, 39 did not meet the requirements of outcome, and two results were duplicated with included studies (Figure 1). A total of 38 studies (6,338 patients) were included in the systematic review, including 27 RCTs and 11 observational studies (Table 1). Twenty-three studies (Presthus et al., 1987; Hietanen, 1991; Nappi et al., 1991; Allain et al., 1993; Lees, 1993; Myllylä et al., 1993; Shoulson, 1993, 1996; Mally et al., 1995; Olanow et al., 1995; Larsen and Boas, 1997; Pålhagen et al., 1998, 2006; Larsen et al., 1999; Lyytinen et al., 2000; Shoulson et al., 2002; Weng et al., 2002; Su et al., 2004; Zhao et al., 2004, 2005; Ye et al., 2014; Mizuno et al., 2017; Tao et al., 2019) reported the outcome which is also reported in at least one other study, and were included in the meta-analysis, and the results of other studies were described in term of outcomes. Selegiline treatment duration ranged from 2 weeks to 7 years.

**Table 1:** Characteristics of included studies.

**Study**	**Country**	**Stage of PD**	**Intervention/control**	**Dosage of selegiline**	**Treatment duration**	**Evaluation date**	**Outcomes**
**Randomized controlled trials**							
**Selegiline monotherapy vs. placebo monotherapy**							
Allain et al. (1993)	France	H&Y score < 2.5	Selegiline:48; Placebo:45	5 mg bid	3 m	1 m 3 m	➀ ➁ ➂ ➃ ➄ ➆
Dalrymple-Alford et al. (1995)	New Zealand	All stages	Selegiline:9; Placebo:11	5 mg bid	4 m	2 m	➀ ➁ ➂
Hietanen (1991)	Finland	All stages	Selegiline:9; Placebo:9	30 mg qd	3 m	3 m	➄
Mally et al. (1995)	UK	H&Y stages I to III	Selegiline:10; Placebo:10	10 mg/d	6 w	1 w 2 w 3 w 1 m 5 w 6 w	➀ ➁ ➂ ➃
Mizuno et al. (2017)	Japan	H&Y stages I to III, UPDRS part III scores 10 points or greater	Selegiline:139; Placebo:140	5 mg bid	3 m	3 m	➀ ➁ ➂ ➆
Myllylä et al. (1993)	Finland	H&Y stages I to III	Selegiline:27; Placebo:25	5 mg bid	12 m	3 w 2 m 4 m 8 m 12 m	➆
Pålhagen et al. (1998)	Sweden	Early stage of PD	Selegiline:81; Placebo:76	10 mg/d	7 y	6 m 12 m	➀ ➁ ➂ ➃ ➆
Shoulson (1996)	America	All stages	Selegiline:189; Placebo:121	5 mg bid	18 m	1 m 4 m	➂ ➆
Shoulson et al. (2002)	America	All stages	Selegiline:191; Placebo:177	10 mg/d	2 y	1 m 3 m 9 m 15 m 21 m	➀ ➁ ➂ ➃ ➆
Su et al. (2004)	China	H&Y stages I to III	Selegiline:71; Placebo:72	5 mg bid	3 m	1 m 3 m	➂ ➆
Weng et al. (2002)	China	All stages	Selegiline:20; Placebo:20	5 mg bid	8 w	1 w 2 w 1 m 6 w 2 m	➅ ➆
Zhao et al. (2005)	China	H&Y stages I or II	Selegiline:12; Placebo:13	0.2 mg/d	13 m	6 m 13 m	➃ ➆
**Selegiline combined with other treatment vs. placebo combined with other treatment**							
Larsen and Boas (1997)	Denmark	H&Y stages I to III	Selegiline + levodopa:73; Placebo + levodopa:81	10 mg/d	5 y	3 m 12 m 24 m 36 m 48 m 54 m	➂ ➃
Larsen et al. (1999)	Norway	H&Y stages I to III	Selegiline + levodopa:73; Placebo + levodopa:81	10 mg qd	5 y	3 m 60 m	➂ ➃ ➆
Lees (1993)	UK	All stages	Selegiline + levodopa-benserazide:271; Levodopa-benserazide:249	5 mg bid	12 m	12 m	➅ ➆
Nappi et al. (1991)	Italy	H&Y stages I to III	Selegiline + lisuride:10; Placebo + lisuride:10	5 mg bid	3 m	1 m 2 m 3 m	➅
Olanow et al. (1995)	America	H&Y stages I to III	Selegiline + sinemet/bromocriptine:52; Placebo + sinemet/bromocriptine:49	10 mg/d	12 m	3 m 12 m 14 m	➁ ➂ ➃
Presthus et al. (1987)	Norway	All stages	Selegiline + Madopar:15; Placebo + Madopar:15	5 mg bid	6 w	6 w	➅
**Selegiline monotherapy vs. therapeutic drug monotherapy**							
Caraceni et al. (1992)	Italy	All stages	Selegiline:157; levodopa:159; lisuride:82; bromocriptine:77	10 mg/d	3 y	2 m	➀ ➁ ➂
Caraceni and Musicco (2001)	Italy	All stages	Selegiline:155; Levodopa:156; Dopamine agonists:162	10 mg/d	Average 3 y	Every 2 m	➂
Zhao et al. (2004)	China	H&Y stages I to II	Selegiline:11; Levodopa-benserazide:11; Trihexyphenidyl:11	10 mg/d	13 m	6 m 13 m	➃ ➆
**Selegiline combined with other treatment vs. therapeutic drug combined with other treatment**							
Lyytinen et al. (2000)	Finland	All stages	Selegiline + levodopa/DDC:16; Entacapone + levodopa/DDC:16	10 mg/d	2 w	2 w	➂ ➆
Ye et al. (2014)	China	All stages	Selegiline + levodopa:54; Pramipexole + levodopa:54	5 mg qd	8 w	2 w 1 m 2 m	➃ ➆
**Others**							
Ahmadiahangar et al. (2005)	Iran	All stages	Selegiline + levodopa and artan:25; Levodopa and artan:18; Bromocriptine + levodopa and artan:34	5 mg bid	3 y	3 y	➃
Frankel et al. (1989)	UK	All stages	Selegiline:12	0,10,20,30,40 mg/d	15 w	3 w 6 w 9 w 12 w 15 w	➃
Pålhagen et al. (2006)	Sweden	Early stage of PD	Selegiline or + levodopa:71; Placebo or + levodopa:69	10 mg/d	7 y	12 m 48 m 60 m	➁ ➂ ➃ ➆
Shoulson (1993)	America	H&Y stages I or II	Selegiline or + tocopherol:399; Placebo or + tocopherol:401	10 mg/d	24 m	1 m 3 m	➀ ➁ ➂ ➃ ➄ ➆
**Observational studies**							
**Selegiline monotherapy vs. therapeutic drug monotherapy**							
Cereda et al. (2017)	Italy	All stage	Selegiline:85; Rasagiline:85	5, 10 mg/d	36 m	36 m	➀ ➁ ➂ ➃
Tao et al. (2019)	China	All stages	Selegiline:250; Pramipexole:250	10 mg/d	36 m	36 m	➂ ➆
**Before-after comparative analysis**							
Chouza et al. (1989)	Uruguay	All stages	Selegiline:13	5 mg bid	4 m	1 m 2 m 3 m 4 m	➅
Djaldetti et al. (2002)	Israel	All stages	Selegiline:15	NA	1 m	1 m	➁ ➂ ➃
Iijima et al. (2017)	Japan	H&Y stages II to III	Selegiline:14	Average dose: 4.0 mg/d	3 m	3 m	➂
LeWitt et al. (1993)	America	H&Y score ≤ 2.5	Selegiline:20	5 mg bid	1 m	1 m	➁ ➂
Li (2004)	China	All stages	Selegiline:9	10 mg/d	3 m	3 m	➂ ➄
Mizuno et al. (2010)	Japan	All stages	Selegiline:691	5.29 ± 2.03 mg/d	7 y; 16 w	7 y 16 w	➂
Mizuno et al. (2019)	Japan	Early stage of PD	Selegiline:134	5 mg bid	56 w	Every 4 weeks	➀ ➁ ➂ ➃ ➆
Ruggieri et al. (1986)	Italy	All stages	Selegiline + levodopa:76	5 mg bid	35 d	10 d 17 d 38 d 45 d	➅ ➆
Wei and Li (2018)	China	All stages	Selegiline or + levodopa:48	5 mg bid	3 m	1 m 3 m	➂ ➄ ➆

Outcomes: ➀ Mental UPDRS; ➁ Activities of daily living UPDRS; ➂ Motor UPDRS; ➃ Total UPDRS; ➄ HAMD score; ➅ WRS score; ➆ adverse events.

PD, Parkinson's disease; H&Y, Hoehn-Yahr stages; UPDRS, Unified Parkinson's Disease Rating Scale; HAMD, Hamilton Depression Rating Scale; WRS, Webster Rating Scale; NA, not available; d, day; w, week; m, month; y, year.

### Research quality evaluation

**Figure 2:**
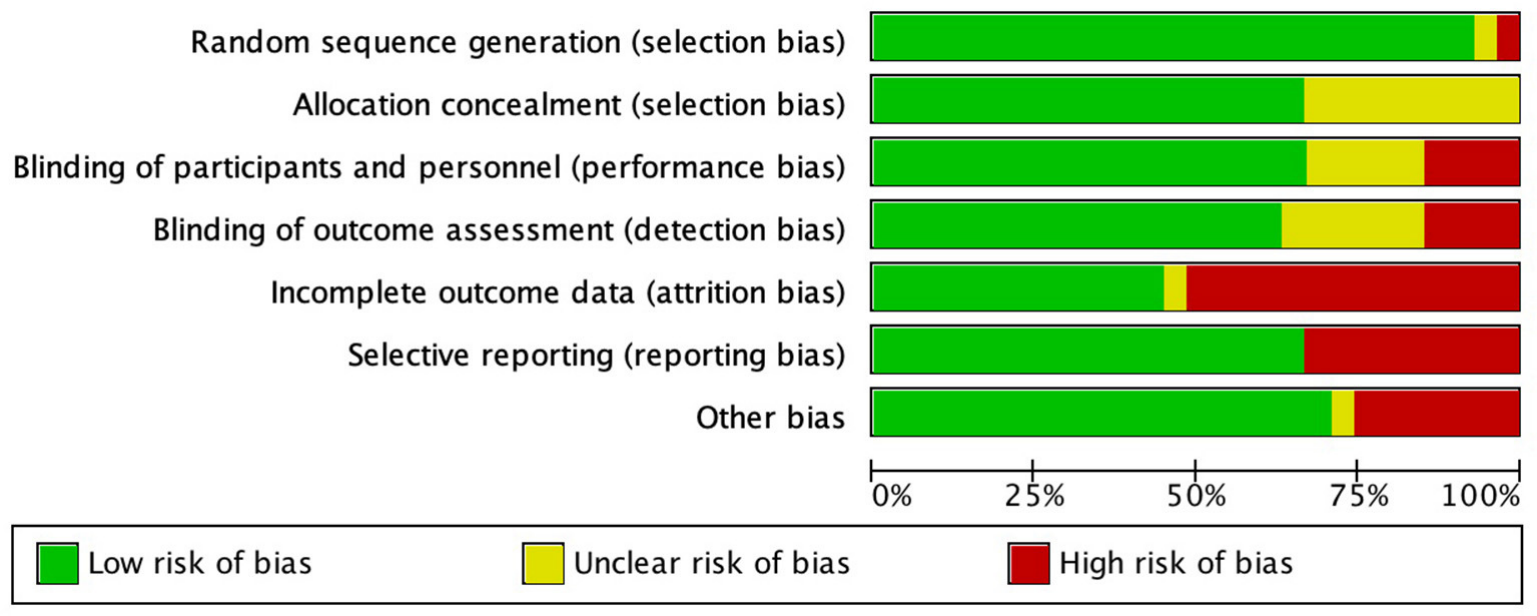
Summary of risk of bias assessment for randomized controlled trials.

Risk of bias analysis of included RCTs was showed in Figure 2. The incompleteness of result information and selective reports were the main reasons for risk of bias. Table 2 showed the results of risk of bias analysis of included cohort studies. The highest quality score was 8 points, while the lowest was 6 points. In general, the risks of bias were moderate in seven studies and low in three studies. The risk of bias for one case control study (Cereda et al., 2017) was low, and the NOS score was 9 points.

**Table 2:** Risk of bias of cohort studies.

**Studies**	**Total NOS scores**	**Selection**				**Comparability**		**Outcomes**		
		**Representativeness of the intervention cohort**	**Non-exposed cohort drawn from the same community as the exposed cohort**	**Ascertainment of exposure from a secure record**	**Demonstration that outcome of interest not present at start of study**	**Cohorts comparable on important factors** ^a^	**Cohorts comparable on other factors** ^b^	**Assessment of outcome of record linkage or independent blind assessment**	**Follow-up long enough for outcomes to occur**	**Complete accounting for cohorts**
Chouza et al. (1989)	7	Y	Y	Y	N^1^	N^2^	Y	Y	Y	Y
Djaldetti et al. (2002)	6	Y	Y	Y	N^1^	Y	Y	N^3^	Y	N^4^
Iijima et al. (2017)	8	Y	Y	Y	N^1^	Y	Y	Y	Y	Y
LeWitt et al. (1993)	7	Y	Y	Y	N^1^	Y	N^5^	Y	Y	Y
Li (2004)	8	Y	Y	Y	N^1^	Y	Y	Y	Y	Y
Mizuno et al. (2010)	6	Y	Y	Y	N^1^	Y	N^6^	Y	Y	N^4^
Mizuno et al. (2019)	7	Y	Y	Y	N^1^	Y	Y	Y	Y	N^4^
Ruggieri et al. (1986)	6	Y	Y	Y	N^1^	N^2^	N^5^	Y	Y	Y
Tao et al. (2019)	7	Y	Y	Y	N^1^	N^2^	Y	Y	Y	Y
Wei and Li (2018)	8	Y	Y	Y	N^1^	Y	Y	Y	Y	Y

Y, related content conforms to this item and the cell with Y was painted to green; N, related content does not conform to this item and the cell with N was painted to red. ^a^Important factors are treatment time of selegiline, doses and drug combination. ^b^Other factors are age, sex. ^1^Outcome measures were evaluated before selegiline administration. ^2^Drug combination has not been compared. ^3^Not described. ^4^More than 20% were lost to follow-up. ^5^Other factors have not been compared. ^6^The ages of the two groups are incomparable.

NOS, Newcastle–Ottawa scale.

### Efficacy

#### UPDRS score

**Figure 3:**
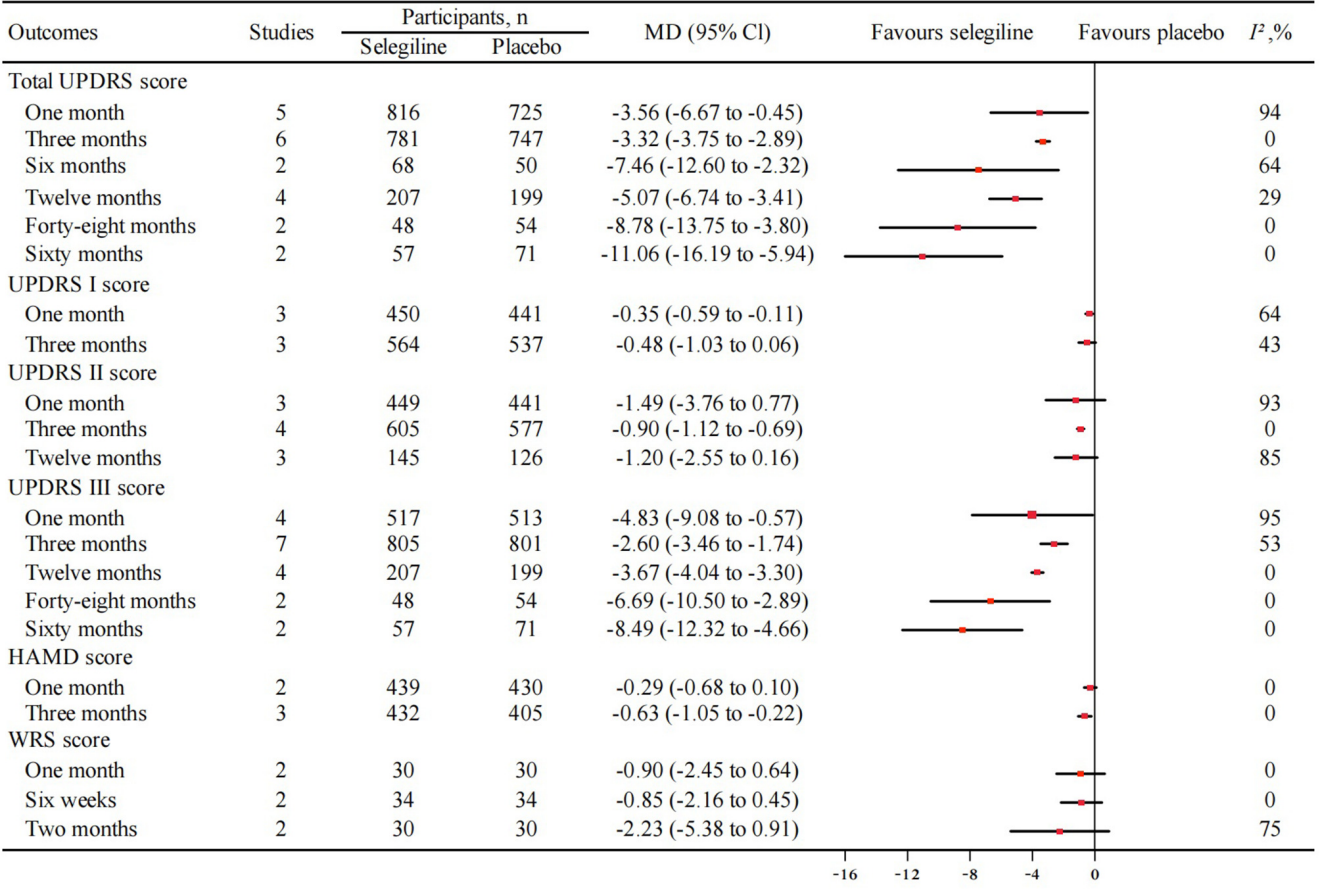
Meta-analysis of improvement in total UPDRS, UPDRS I, UPDRS II, UPDRS III, HAMD, WRS score between selegiline and placebo. UPDRS, unified Parkinson's disease rating scale; UPDRS I, mental score; UPDRS II, activities of daily living score; UPDRS III, motor score; HAMD, Hamilton depression rating scale; WRS, webster rating scale; MD, mean difference; 95% CI, 95% confidence interval.

A total of 15 RCTs reported the change in total UPDRS score. Eleven RCTs comparing selegiline with placebo were included in the meta-analysis, and showed selegiline significantly improved the total UPDRS score with an increasing tread after 1 month (MD −3.56, 95% CI −6.67 to −0.45, *P* = 0.02, *I*^2^ = 94%), 3 months (MD −3.32, 95% CI −3.75 to −2.89, *P* < 0.00001, *I*^2^ = 0%), 6 months (MD −7.46, 95% CI −12.60 to −2.32, *P* = 0.09, *I*^2^ = 64%), 12 months (MD −5.07, 95% CI −6.74 to −3.41, *P* < 0.00001, *I*^2^ = 29%), 48 months (MD −8.78, 95% CI −13.75 to −3.80, *P* = 0.0005, *I*^2^ = 0%), and 60 months (MD −11.06, 95% CI −16.19 to −5.94, *P* < 0.0001, *I*^2^ = 0%) of treatment (Figure 3, Supplementary Figure 1A). Further subgroup analysis showed that the total UPDRS score of selegiline monotherapy and in combination with an PD treatment also tended to improve over time compared with placebo (Supplementary Figure 2). Three RCTs reported the comparison between selegiline and the other active controls, showing selegiline was better than trihexyphenidyl, pramipexol, and bromocriptine and inferior to levodopa-benserazide in improving total UPDS score during the study period (Zhao et al., 2004; Ahmadiahangar et al., 2005; Ye et al., 2014). One observational study showed selegiline was similar with resagiline in improving UPDRS score (Cereda et al., 2017). Frankel et al. (1989) found high doses of selegiline was not superior to conventional doses in improving UPDRS score.

There were 8, 10 and 15 RCTs reporting the UPDRS I, II and III score respectively, with 4, 7 and 10 reporting the comparison between selegiline and placebo included in the meta-analysis. With increasing treatment duration, there was a trend of increasing improvement by selegiline in the change of UPDRS I (after 1 month: MD −0.35, 95% CI −0.59 to −0.11, *P* = 0.005, *I*^2^ = 64%; after 3 months: MD −0.48, 95% CI −1.03 to 0.06, *P* = 0.08, *I*^2^ = 43%) (Figure 3, Supplementary Figure 1B), UPDRS II (after 1 month: MD −1.49, 95% CI −3.76 to 0.77, *P* = 0.20, *I*^2^ = 93%; after 3 months: MD −0.90, 95% CI −1.12 to −0.69, *P* < 0.00001, *I*^2^ = 0%; after 12 months: MD −1.20, 95% CI −2.55 to 0.16, *P* = 0.08, *I*^2^ = 85%) (Figure 3, Supplementary Figure 1C), and UPDRS III score (after 1 month: MD −4.83, 95% CI −9.08 to −0.57, *P* = 0.03, *I*^2^ = 95%; after 3 months: MD −2.60, 95% CI −3.46 to −1.74, *P* < 0.00001, *I*^2^ = 53%; after 12 months: MD −3.67, 95% CI −4.04 to −3.30, *P* < 0.00001, *I*^2^ = 0%; after 48 months: MD −6.69, 95% CI −10.50 to −2.89, *P* = 0.0006, *I*^2^ = 0%; after 60 months: MD −8.49, 95% CI −12.32 to −4.66, *P* < 0.0001, *I*^2^ = 0%) (Figure 3, Supplementary Figure 1D).

Three RCTs reported the comparison between selegiline and placebo at the other follow-up period. Selegiline significantly improved UPDRS I at 2 months and 6 months, but not at 12 months and an average of 2 years (Dalrymple-Alford et al., 1995; Pålhagen et al., 1998; Shoulson et al., 2002). Selegiline significantly improved UPDRS II and III score during an average of 2 years of follow-up, but not at 2 months (Dalrymple-Alford et al., 1995; Shoulson et al., 2002). Three RCTs reported the comparison between selegiline and the other active controls, showing no statistical difference among levodopa, bromocriptine, lisuride, entacapone and selegiline in improving UPDRS I and III score (Caraceni et al., 1992; Lyytinen et al., 2000; Caraceni and Musicco, 2001). UPDRS II score was significantly improved among patients treated with selegiline compared with patients treated with levodopa, bromocriptine, and lisuride (Caraceni et al., 1992). One observational study showed the improvement in UPDRS III was higher for pramipexole than selegiline (Tao et al., 2019).

Seven observational studies analyzed pre-administration and post-administration UPDRS score change with selegiline. Four studies noted significant improvements in UPDRS III after 3 months of selegiline treatment (Li, 2004; Mizuno et al., 2010; Iijima et al., 2017; Wei and Li, 2018). LeWitt et al. (1993) found no significant difference in change of UPDRS II and UPDRS III from baseline after 1 month of selegiline treatment. Mizuno et al. (2019) reported selegiline significantly reduced total UPDRS score from week 4 to week 56. Similar improvements were also found in UPDRS II and UPDRS III scores. However, there was no significant decrease in UPDRS I score at all time points. Djaldetti et al. (2002) indicated there was no significant change in total UPDRS, UPDRS II and UPDRS III score after selegiline withdrawal for 1 month.

#### HAMD score

Three RCTs which reported the change of HAMD score were included in the meta-analysis. There was also a trend in improving HAMD score with increasing treatment durations (after 1 month: MD −0.29, 95% CI −0.68 to 0.10, *P* = 0.15, *I*^2^ = 0%; after 3 months: MD −0.63, 95% CI −1.05 to −0.22, *P* = 0.003, *I*^2^ = 0%) (Figure 3, Supplementary Figure 3). Three observational studies analyzed pre-administration and post-administration HAMD score change with selegiline, showing significant improvement after 3 months of treatment (Li, 2004; Iijima et al., 2017; Wei and Li, 2018).

#### WRS score

Four RCTs reported the change of WRS score and three were included in the meta-analysis. The results showed a trend in improving WRS score with increasing treatment durations, but no statistical difference between selegiline and placebo (after 1 month: MD −0.90, 95% CI −2.45 to 0.64, *P* = 0.25, *I*^2^ = 0%; after 6 weeks: MD −0.85, 95% CI −2.16 to 0.45, *P* = 0.20, *I*^2^ = 0%; after 2 months: MD −2.23, 95% CI −5.38 to 0.91, *P* = 0.16, *I*^2^ = 75%) (Figure 3, Supplementary Figure 4). Similarly, Lees (1993) showed no significant difference in change of WRS score was found between selegiline and placebo during 12 months of follow up. Two observational studies analyzed pre-administration and post-administration WRS score change with selegiline. Chouza et al. (1989) showed a mild decrease but no significant change in WRS score after 4 months of selegiline treatment, while Ruggieri et al. (1986) demonstrated significant decrease within 45 days.

### Safety

#### General information of adverse events

A total of 20 studies described the incidence of adverse events and respectively described adverse events of neuropsychiatric disorders, musculoskeletal and connective tissue disorders, cardiovascular disorders, gastrointestinal disorders, liver diseases, and skin reaction which were mentioned in the instructions of selegiline. The details were exhibited in Supplementary Table 1.

**Figure 4:**
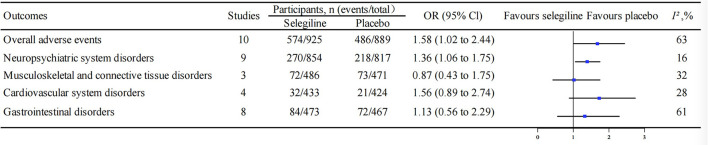
Comparison of the incidence for overall adverse events and those stratified by disorders between selegiline and placebo. OR, odds ratio; 95% CI, 95% confidence interval.

For the overall adverse events, ten studies including 1,814 individuals were included in the meta-analysis and the results proved that the overall incidence of adverse events with selegiline was higher than that with placebo (rate: 62.1% vs. 54.7%, OR 1.58, 95% CI 1.02 to 2.44, *P* = 0.04, *I*^2^ = 63%) (Figure 4). We did not find selegiline with significant difference in overall adverse event with the following active controls (Entacapone: OR 2.06, 95% CI 0.43 to 9.80, *P* = 0.36; Pramipexole: OR 0.19, 95% CI 0.01 to 2.59, *P* = 0.21; Trihexyphenidyl: OR 0.05, 95% CI 0.00 to 1.09, *P* = 0.06) (Supplementary Figure 5A).

#### Adverse events on various disorders

Twelve studies reported data on adverse events of neuropsychiatric disorders. The results indicated that the selegiline had higher possibility to encounter neuropsychiatric disorders than the placebo (rate: 31.6% vs. 26.7%, OR 1.36, 95% CI 1.06 to 1.75, *P* = 0.02, *I*^2^ = 16%) (Figure 4). There was a significant increase in neuropsychiatric disorders at 12 months of selegiline treatment compared with placebo (OR 1.49, 95% CI 1.06 to 2.10, *P* = 0.02, *I*^2^ = 0%). However, the significant trend of the increase in adverse events over time was not found (Supplementary Figure 6). Selegiline treatment for 60 months did not significantly increase neuropsychiatric adverse events compared with placebo (OR 1.81, 95% CI 0.96 to 3.42, *P* = 0.07, *I*^2^ = 0%). Further analysis showed that selegiline had more adverse reactions such as vertigo, headache, hallucination, and fatigue than placebo, but the results were not statistically different (Supplementary Figure 7). In addition, selegiline did not increase the incidence of adverse events of neuropsychiatric disorders compared with the following active controls (Entacapone: OR 1.50, 95% CI 0.33–6.77, *P* = 0.60; Pramipexole: OR 0.80, 95% CI 0.52–1.24, *P* = 0.32; Trihexyphenidyl: OR 6.05, 95% CI 0.26–142.06, *P* = 0.26) (Supplementary Figure 5B).

Four studies described data on adverse events in musculoskeletal and connective tissue disorders. The meta-analysis results showed no significant difference in musculoskeletal and connective tissue disorders between selegiline and placebo (rate: 14.8% vs. 15.5%, OR 0.87, 95% CI 0.43–1.75, *P* = 0.69, *I*^2^ = 32%) (Figure 4). Patients with selegiline had less musculoskeletal and connective tissue disorders than with pramipexole (OR 0.10, 95% CI 0.03–0.29, *P* < 0.0001) (Supplementary Figure 5C).

Six studies reported adverse events of cardiovascular disorders and were included in the meta-analysis. The results reflected that there was no significant difference about cardiovascular adverse events in selegiline group compared with placebo group (rate: 7.4% vs. 5.0%, OR 1.56, 95% CI 0.89 to 2.74, *P* = 0.12, *I*^2^ = 28%) (Figure 4) and entacapone group (OR 3.00, 95% CI 0.48 to 18.93, *P* = 0.24). Patients with selegiline had a higher incidence of cardiovascular adverse events than with pramipexole (OR 5.26, 95% CI 1.50 to 18.39, *P* = 0.009) (Supplementary Figure 5D).

A total of 11 studies reported the gastrointestinal adverse events. The meta-analysis results showed that the incidence of gastrointestinal adverse events in selegiline group was not significantly different from that in placebo group (rate: 17.8% vs. 15.4%, OR 1.13, 95% CI 0.56–2.29, *P* = 0.74, *I*^2^ = 61%) (Figure 4), entacapone group (OR 1.50, 95% CI 0.21–10.65, *P* = 0.69) and pramipexole group (OR 1.22, 95% CI 0.84–1.77, *P* = 0.30). Patients with selegiline had less gastrointestinal adverse events than with trihexyphenidyl (OR 0.03, 95% CI 0.00–0.56, *P* = 0.02) (Supplementary Figure 5E).

#### Sensitivity analysis

One month of total UPDRS, UPDRS II, and UPDRS III score had high heterogeneity. Different from the other studies, Mally et al. showed selegiline had a more significant improvement in total UPDRS, UPDRS II, and UPDRS III score. Therefore, the sensitivity analysis was performed after eliminating this study. The heterogeneity was reduced (total UPDRS: MD −1.14, 95% CI −2.91 to 0.63, *P* = 0.21, *I*^2^ = 81%; UPDRS II: MD −0.46, 95% CI −0.77 to −0.14, *P* = 0.005, *I*^2^ = 0%; UPDRS III: MD −2.97, 95% CI −6.63 to 0.68, *P* = 0.11, *I*^2^ = 78%). The results showed no statistical difference for total UPDRS and UPDRS III, while an improvement trend was still identified.

## Discussion

This is the first systematic review and meta-analysis that focus on the efficacy and safety of selegiline by different treatment durations. We found that selegiline significantly improved the total UPDRS score and the effect increased as the treatment duration increased. A similar trend was also found from the point estimates in UPDRS I, II, III, HAMD and WRS score. Selegiline had a higher risk of incurring adverse events, with the excess adverse events mainly manifested as neuropsychiatric disorders. The statistically difference in the overall adverse events between selegiline and active controls was not found.

Previous studies have confirmed the efficacy of selegiline in patients with PD. A network meta-analysis indicated that selegiline exhibited a significant improvement in UPDRS II, UPDRS III and total UPDRS scores (Zhuo et al., 2017). A meta-analysis pointed out that selegiline plus levodopa combination therapy significantly improved total UPDRS, UPDRS I, UPDRS II, UPDRS III scores and WRS score compared with levodopa monotherapy (Jiang et al., 2020). And a multiple treatment comparison meta-analyses found selegiline to be efficient in changing UPDRS score compared to placebo (Binde et al., 2020). However, all the above meta-analyses did not consider the effect of medication duration on efficacy. Consistent with previous studies, this study showed an improvement in total UPDRS, UPDRS III scores and HAMD score with selegiline. In addition, this study found that the effect gradually increased in 3, 12, 48, and 60 months, which further confirmed the efficacy of long-term use of selegiline. Moreover, there was great heterogeneity in the 1 month of UPDRS score, and the stability of the results was poor, which may be affected by combined factors of drugs, dosage and population. The result was more robust after 3 months of medication. The improvement in total UPDRS scores was found to be slightly lower at the 6 months measurement compared with that at the 3 months measurement, which may be influenced by disease progression. In addition, different from studies conducted by Zhang et al. (2015) and Jiang et al. (2020), this study did not find selegiline significantly improving WRS score at 1 month, 6 weeks or 2 months of treatment, which can be attributed to different inclusion and exclusion criteria. We excluded studies from non-core journals to help improve the quality of included researches.

Previous studies have shown inconsistent results regarding the safety of selegiline. Jiang et al. (2020) found selegiline plus levodopa compared with levodopa monotherapy was not associated with significantly more adverse events, and Macleod et al. (2005) also found that selegiline was not associated with higher rate of withdrawals due to adverse events. However, Jost et al. (2012) found that selegiline was associated with higher rate of study discontinuation due to adverse effects compared with placebo groups. This study found that selegiline was associated with higher incidence of any adverse events than those of placebo, but such association was not detected when comparing with entacapone, pramipexole or trihexyphenidyl. Among the comparison between selegiline and placebo in the safety outcome, we found that selegiline was associated with higher incidence of adverse events of neuropsychiatric disorders, but not in the musculoskeletal and connective tissue disorders, cardiovascular disorders or gastrointestinal disorders respectively. Selegiline was related to higher rate of fatigue, headache and somnolence and lower rate of anxiety and depression compared with placebo, which supported the findings of previous studies (Tan et al., 2022; Tsuboi et al., 2022). In addition, most studies we included did not report a course of adverse reactions with selegiline, and only one study reported an adverse event in a patient with hallucination that occurred after 2 weeks of medication, and symptoms improved after dose reduction (Weng et al., 2002). We did not find significant increase in neuropsychiatric adverse events with selegiline over time, which may be limited by the included studies. As there is no original study reporting the adverse events in a shorter timeframe, we cannot capture such a trend of increasing relative risk of adverse events comparing selegiline with placebo. In addition, there is limited studies reporting the adverse events in a much shorter or longer timeframe, limiting the power of our synthesis to show significant results.

There are several strengths in our study. Firstly, the update of RCTs was more comprehensive in our study. We included all populations of PD who used selegiline and did not restrict the use of drugs in the control group, thus covering a more comprehensive range of relevant original studies and providing more information than previous studies, including temporal correlation of effect and results compared with active controls. Secondly, we also included observational studies to augment current evidence, which was omitted from the previous studies. The before-after comparative of efficacy from observational studies provided an additional indication of how the effect of selegiline over time was affected by disease progression. Thirdly, we evaluated the occurrence of adverse events into multiple different disorders for more detailed observation, and found more adverse events of neuropsychiatric disorders with selegiline than with placebo.

However, this meta-analysis still has some limitations. The temporal association found in our studies may be dominated by the trends from the RCTs which reported the outcomes at different timings of measurement. As only limited studies were included, heterogeneity in the results cannot be further explored. In addition, the effect of disease stage, course of disease and diet on the selegiline's efficacy and safety over time were difficult to determine in this study, but could have influenced the results. To lay a solid foundation for carrying out high quality systematic evaluation and health economics evaluation in the future (Yi et al., 2022), we expect higher quality and more convincing clinical studies.

## Conclusion

Selegiline was found to be effective in improving total UPDRS score and the effect increased with the treatment duration. The trend was also found in UPDRS I, II, III, HAMD and WRS score. As for safety, selegiline had higher risk of incurring any adverse events than placebo, with the excess adverse events mainly manifested as neuropsychiatric disorders. Further analysis is required to confirm the temporal correlation of efficacy and safety of selegiline.

## Data availability statement

The original contributions presented in the study are included in the article/Supplementary material, further inquiries can be directed to the corresponding authors.

## Author contributions

Z-HL, J-RL, and Y-FL screened the studies through full text reading. KW and X-YL had full access to all the data in the manuscript and take responsibility for the integrity of the data and the accuracy of the data analysis. KW and Z-HL drafting of the manuscript. KW, Z-MY, Z-HL, J-JH, J-XL, and J-WZ critical revision of the manuscript for important intellectual content. KW contributed to the statistical analysis. Z-MY contributed to the concept, design, supervision, and funding. All authors contributed to the data acquisition, analysis, and interpretation of data.

## References

[B1] Ahmadiahangar A. Sadraie A. Vaghefi S. Ramesani M. (2005). Comparison between bromocriptine and selegiline in treatment of Parkinson. Daru. 13, 23–27.

[B2] Allain H. Pollak P. Neukirch H. C. (1993). Symptomatic effect of selegiline in *de novo* Parkinsonian patients. The French Selegiline Multicenter. Trial. Mov. Disord. 8, S36–40. 10.1002/mds.870080508 8302306

[B3] Armstrong M. J. Okun M. S. (2020). Diagnosis and treatment of Parkinson disease: a review. JAMA. 323, 548–560. 10.1001/jama.2019.22360 32044947

[B4] Binde C. D. Tvete I. F. Gåsemyr J. I. Natvig B. Klemp M. (2020). Comparative effectiveness of dopamine agonists and monoamine oxidase type-B inhibitors for Parkinson's disease: a multiple treatment comparison meta-analysis. Eur. J. Clin. Pharmacol. 76, 1731–1743. 10.1007/s00228-020-02961-6 32710141 PMC7661406

[B5] Caraceni T. Musicco M. (2001). Levodopa or dopamine agonists, or deprenyl as initial treatment for Parkinson's disease. A randomized multicenter study. Parkinsonism Relat. Disord. 7, 107–114. 10.1016/S1353-8020(00)00023-7 11248591

[B6] Caraceni T. Musicco M. Gasparini M. Beghi E. (1992). A multicenter Italian randomised study on early treatment of Parkinson disease: comparison of L-dopa, l-deprenyl and dopaminoagonists. Study design and short term results. The Italian Parkinson Study Group. Ital. J. Neurol. Sci. 13, 735–739. 10.1007/BF02229158 1362394

[B7] Cereda E. Cilia R. Canesi M. Tesei S. Mariani C. B. Zecchinelli A. L. . (2017). Efficacy of rasagiline and selegiline in Parkinson's disease: a head-to-head 3-year retrospective case-control study. J. Neurol. 264, 1254–1263. 10.1007/s00415-017-8523-y 28550482 PMC5570795

[B8] Chai J. Ho R. C. M. (2021). “Hamilton Rating Scale for depression,” in: *Encyclopedia of Gerontology and Population Aging*, eds D. Gu, and M. E. Dupre (Cham: Springer), 2246–2248. 10.1007/978-3-030-22009-9_826

[B9] Chouza C. Aljanati R. Scaramelli A. De Medina O. Caamaño J. L. Buzo R. . (1989). Combination of selegiline and controlled release levodopa in the treatment of fluctuations of clinical disability in parkinsonian patients. Acta Neurol. Scand. Suppl. 126, 127–137. 10.1111/j.1600-0404.1989.tb01792.x 2515718

[B10] Dalrymple-Alford J. C. Jamieson C. F. Donaldson I. M. (1995). Effects of selegiline (deprenyl) on cognition in early Parkinson's disease. Clin. Neuropharmacol. 18, 348–359. 10.1097/00002826-199508000-00007 8665548

[B11] Djaldetti R. Ziv I. Melamed E. (2002). The effect of deprenyl washout in patients with long-standing Parkinson's disease. J. Neural. Transm. 109, 797–803. 10.1007/s007020200066 12111469

[B12] Fahn S. Elton R. L. and U. P. D. R.S. Program Members (1987). “Unified Parkinson's disease rating scale,” in Recent Developments in Parkinson's Disease, eds S. Fahn, C. D. Marsden, M. Goldstein, and D. B. Calne (New Jersey: Macmillan Healthcare Information), 153–163, 293–304.

[B13] Frankel J. P. Kempster P. A. Stibe C. M. Eatough V. M. Nathanson M. Lees A. J. . (1989). A double-blind, controlled study of high-dose L-deprenyl in the treatment of Parkinson's disease. Clin. Neuropharmacol. 12, 448–451. 10.1097/00002826-198910000-00010 2514981

[B14] GBD 2016 Neurology Collaborators (2018). Global, regional, and national burden of Parkinson's disease, 1990–2016: a systematic analysis for the Global Burden of Disease Study 2016. Lancet Neurol. 17, 939–953. 10.1016/S1474-4422(18)30499-X 30287051 PMC6191528

[B15] Ginanneschi A. Degl'Innocenti F. Magnolfi S. Maurello M. T. Catarzi L. Marini P. . (1988). Evaluation of Parkinson's disease: reliability of three rating scales. Neuroepidemiology 7, 38–41. 10.1159/000110159 3340268

[B16] Grimes D. Fitzpatrick M. Gordon J. Miyasaki J. Fon E. A. Schlossmacher M. . (2019). Canadian guideline for Parkinson disease. CMAJ. 191, 989–1004. 10.1503/cmaj.181504 31501181 PMC6733687

[B17] Hamilton M. (1960). A rating scale for depression. J. Neurol. Neurosurg. Psychiatry 23, 56–62. 10.1136/jnnp.23.1.56 14399272 PMC495331

[B18] Hietanen M. H. (1991). Selegiline and cognitive function in Parkinson's disease. Acta Neurol. Scand. 84, 407–410. 10.1111/j.1600-0404.1991.tb04978.x 1776388

[B19] Higgins J. P. Altman D. G. Gøtzsche P. C. Jüni P. Moher D. Oxman A. D. . (2011). The Cochrane Collaboration's tool for assessing risk of bias in randomised trials. BMJ 343, d5928. 10.1136/bmj.d5928 22008217 PMC3196245

[B20] Higgins J. P. Thompson S. G. (2002). Quantifying heterogeneity in a meta-analysis. Stat. Med. 21, 1539–1558. 10.1002/sim.1186 12111919

[B21] Iijima M. Mitoma H. Uchiyama S. Kitagawa K. (2017). Long-term monitoring gait analysis using a wearable device in daily lives of patients with Parkinson's disease: the efficacy of selegiline hydrochloride for gait disturbance. Front. Neurol. 8, 542. 10.3389/fneur.2017.00542 29114238 PMC5660685

[B22] Ives N. J. Stowe R. L. Marro J. Counsell C. Macleod A. Clarke C. E. . (2004). Monoamine oxidase type B inhibitors in early Parkinson's disease: meta-analysis of 17 randomised trials involving 3,525 patients. BMJ 329, 593. 10.1136/bmj.38184.606169.AE 15310558 PMC516655

[B23] Jiang D. Q. Li M. X. Jiang L. L. Chen X. B. Zhou X. W. (2020). Comparison of selegiline and levodopa combination therapy vs. levodopa monotherapy in the treatment of Parkinson's disease: a meta-analysis. Aging Clin. Exp. Res. 32, 769–779. 10.1007/s40520-019-01232-4 31175606

[B24] Jost W. Friede M. Schnitker J. (2012). Indirect meta-analysis of randomised placebo-controlled clinical trials of rasagiline and selegiline in the symptomatic treatment of Parkinson's disease. Basal Ganglia. 2, 17–S26. 10.1016/j.baga.2012.05.006

[B25] Larsen J. P. Boas J. (1997). The effects of early selegiline therapy on long-term levodopa treatment and parkinsonian disability: an interim analysis of a Norwegian–Danish 5-year study. Norwegian-Danish Study Group. Mov. Disord. 12, 175–182. 10.1002/mds.870120207 9087975

[B26] Larsen J. P. Boas J. Erdal J. E. (1999). Does selegiline modify the progression of early Parkinson's disease? Results from a 5-year study. The Norwegian-Danish Study Group. Eur. J. Neurol. 6, 539–547. 10.1046/j.1468-1331.1999.650539.x 10457386

[B27] Lees A. J. (1993). Comparisons of therapeutic effects of levodopa, levodopa and selegiline, and bromocriptine in patients with early, mild Parkinson's disease: 3 year interim report. BMJ 307, 469–472. 10.1136/bmj.307.6902.469 8400928 PMC1678739

[B28] LeWitt P. A. Segel S. A. Mistura K. L. Schork M. A. (1993). Symptomatic anti-parkinsonian effects of monoamine oxidase-B inhibition: comparison of selegiline and lazabemide. Clin. Neuropharmacol. 16, 332–337. 10.1097/00002826-199308000-00005 8374913

[B29] Li J. (2004). The Clinical Study of Depression in Parkinson's Disease. Zhejiang University. Available online at: https://kns.cnki.net/KCMS/detail/detail.aspx?dbname=CMFD9904andfilename=2004062140.nh (assessed January 5, 2022).

[B30] Lyytinen J. Kaakkola S. Gordin A. Kultalahti E. Teräväinen H. (2000). Entacapone and selegiline with L-dopa in patients with Parkinson's disease: an interaction study. Parkinsonism Relat. Disord. 6, 215–222. 10.1016/S1353-8020(00)00012-2 10900396

[B31] Macleod A. D. Counsell C. E. Ives N. Stowe R. (2005). Monoamine oxidase B inhibitors for early Parkinson's disease. Cochrane Database Syst. Rev. 2005, Cd004898. 10.1002/14651858.CD004898 PMC8859569 16034956

[B32] Magyar K. (2011). The pharmacology of selegiline. Int. Rev. Neurobiol. 100, 65–84. 10.1016/B978-0-12-386467-3.00004-2 21971003

[B33] Mally J. Kovacs A. B. Stone T. W. (1995). Delayed development of symptomatic improvement by (–)-deprenyl in Parkinson's disease. J. Neurol. Sci. 134, 143–145. 10.1016/0022-510X(95)00240-1 8747857

[B34] Miyasaki J. M. Shannon K. Voon V. Ravina B. Kleiner-Fisman G. Anderson K. . (2006). Quality standards subcommittee of the American academy of neurology. Practice parameter: evaluation and treatment of depression, psychosis, and dementia in Parkinson disease (an evidence-based review): report of the quality standards subcommittee of the American academy of neurology. Neurology. 66, 996–1002. 10.1212/01.wnl.0000215428.46057.3d 16606910

[B35] Mizuno Y. Hattori N. Kondo T. Nomoto M. Origasa H. Takahashi R. . (2017). A Randomized double-blind placebo-controlled phase III trial of selegiline monotherapy for early Parkinson disease. Clin. Neuropharmacol. 40, 201–207. 10.1097/WNF.0000000000000239 28857772 PMC5610558

[B36] Mizuno Y. Hattori N. Kondo T. Nomoto M. Origasa H. Takahashi R. . (2019). Long-term selegiline monotherapy for the treatment of early Parkinson disease. Clin. Neuropharmacol. 42, 123–130. 10.1097/WNF.0000000000000343 31045589

[B37] Mizuno Y. Kondo T. Kuno S. Nomoto M. Yanagisawa N. (2010). Early addition of selegiline to L-Dopa treatment is beneficial for patients with Parkinson disease. Clin. Neuropharmacol. 33, 1–4. 10.1097/WNF.0b013e3181bbf45c 19935410

[B38] Moore J. J. Saadabadi A. (2022). Selegiline [Internet]. Treasure Island, FL: StatPearls Publishing. Available online at: https://www.ncbi.nlm.nih.gov/books/NBK526094/ (assessed May 5, 2022).

[B39] Myllylä V. V. Sotaniemi K. A. Vuorinen J. A. Heinonen E. H. (1993). Selegiline in *de novo* parkinsonian patients: the Finnish study. Mov. Disord. 8, S41–44. 10.1002/mds.870080509 8302307

[B40] Nagatsu T. Sawada M. (2006). Molecular mechanism of the relation of monoamine oxidase B and its inhibitors to Parkinson's disease: possible implications of glial cells. J. Neural. Transm. Suppl. 2006, 53–65. 10.1007/978-3-211-33328-0_7 17447416

[B41] Nappi G. Martignoni E. Horowski R. Pacchetti C. Rainer E. Bruggi P. . (1991). Lisuride plus selegiline in the treatment of early Parkinson's disease. Acta Neurol. Scand. 83, 407–410. 10.1111/j.1600-0404.1991.tb03973.x 1909485

[B42] NICE (2017). Parkinson's Disease in Adults: Diagnosis and Management. London: National Institute for Health and Care Excellence (NICE). 28787113

[B43] Obeso J. A. Stamelou M. Goetz C. G. Poewe W. Lang A. E. Weintraub D. . (2017). Past, present, and future of Parkinson's disease: a special essay on the 200th Anniversary of the Shaking Palsy. Mov. Disord. 32, 1264–1310. 10.1002/mds.27115 28887905 PMC5685546

[B44] Olanow C. W. Hauser R. A. Gauger L. Malapira T. Koller W. Hubble J. . (1995). The effect of deprenyl and levodopa on the progression of Parkinson's disease. Ann. Neurol. 38, 771–777. 10.1002/ana.410380512 7486869

[B45] Pålhagen S. Heinonen E. Hägglund J. Kaugesaar T. Mäki-Ikola O. Palm R. (2006). Selegiline slows the progression of the symptoms of Parkinson disease. Neurology 66, 1200–1206. 10.1212/01.wnl.0000204007.46190.54 16540603

[B46] Pålhagen S. Heinonen E. H. Hägglund J. Kaugesaar T. Kontants H. Mäki-Ikola O. . (1998). Selegiline delays the onset of disability in *de novo* parkinsonian patients. Swedish Parkinson Study Group. Neurology. 51, 520–525. 10.1212/WNL.51.2.520 9710028

[B47] Presthus J. Berstad J. Lien K. (1987). Selegiline (1-deprenyl) and low-dose levodopa treatment of Parkinson's disease. A double-blind crossover trial. Acta Neurol. Scand. 76, 200–203. 10.1111/j.1600-0404.1987.tb03567.x 3120487

[B48] Ramaker C. Marinus J. Stiggelbout A. M. Van Hilten B. J. (2002). Systematic evaluation of rating scales for impairment and disability in Parkinson's disease. Mov. Disord. 17, 867–876. 10.1002/mds.10248 12360535

[B49] Ruggieri S. Denaro A. Meco G. Carta A. Stocchi F. Agnoli A. (1986). Multicenter trial of L-Deprenyl in Parkinson disease. Ital. J. Neurol. Sci. 7, 133–137. 10.1007/BF02230431 3082793

[B50] Shoulson I. (1993). Effects of tocopherol and deprenyl on the progression of disability in early Parkinson's disease. N. Engl. J. Med. 328, 176–183. 10.1056/NEJM199301213280305 8417384

[B51] Shoulson I. (1996). Impact of deprenyl and tocopherol treatment on Parkinson's disease in DATATOP subjects not requiring levodopa. Parkinson Study Group. Ann. Neurol. 39, 29–36. 10.1002/ana.410390106 8572663

[B52] Shoulson I. Oakes D. Fahn S. Lang A. Langston J. W. LeWitt P. . (2002). Impact of sustained deprenyl (selegiline) in levodopa-treated Parkinson's disease: a randomized placebo-controlled extension of the deprenyl and tocopherol antioxidative therapy of parkinsonism trial. Ann. Neurol. 51, 604–612. 10.1002/ana.10191 12112107

[B53] Simon D. K. Tanner C. M. Brundin P. (2020). Parkinson disease epidemiology, pathology, genetics, and pathophysiology. Clin. Geriatr. Med. 36, 1–12. 10.1016/j.cger.2019.08.002 31733690 PMC6905381

[B54] Su N. Wu B. Xu T. (2014). Effectiveness and safety of selegiline in the treatment of Parkinson's disease: a systematic review. Chin. J. Hosp. Pharm. 34, 1206–1212. 10.13286/j.cnki.chinhosppharmacyj.2014.14.1734784871

[B55] Su W. Chen H. Zhang Z. Chen B. Wang L. Sun X. . (2004). A multi-center, randomized, vitamin E controlled and opening clinical trial of selegiline in patients with Parkinson's disease. Chin. J. Neurol. 37, 413–416. 10.3760/j.issn:1006-7876.2004.05.00830704229

[B56] Tábi T. Vécsei L. Youdim M. B. Riederer P. Szöko É. (2020). Selegiline: a molecule with innovative potential. J. Neural. Transm. 127, 831–842. 10.1007/s00702-019-02082-0 31562557 PMC7242272

[B57] Tan Y. Y. Jenner P. Chen S. D. (2022). Monoamine oxidase-B inhibitors for the treatment of Parkinson's disease: past, present, and future. J. Parkinsons Dis. 12, 477–493. 10.3233/JPD-212976 34957948 PMC8925102

[B58] Tao Z. Chen J. Xiao L. Liu C. (2019). Pramipexole vs. selegiline in patients with Parkinson's disease: an effectiveness and safety (EAS) analysis. Iran Red. Crescent. Med. J. 21, e96672. 10.5812/ircmj.96672

[B59] Tsuboi T. Satake Y. Hiraga K. Yokoi K. Hattori M. Suzuki M. . (2022). Effects of MAO-B inhibitors on non-motor symptoms and quality of life in Parkinson's disease: a systematic review. NPJ Parkinsons Dis. 8, 75. 10.1038/s41531-022-00339-2 35697709 PMC9192747

[B60] van den Heuvel L. Evers L. J. W. Meinders M. J. Post B. Stiggelbout A. M. Heskes T. M. . (2021). Estimating the effect of early treatment initiation in Parkinson's disease using observational data. Mov. Disord. 36, 407–414. 10.1002/mds.28339 33107639 PMC7984449

[B61] Webster D. D. (1968). Critical analysis of the disability in Parkinson's disease. Mod. Treat. 5, 257–282. 5655944

[B62] Wei J. Li X. (2018). Therapeutic effect of Selegiline on patients with Parkinson's disease with frozen gait. Chin. J. Integr. Med. Cardio-/Cerebrovasc. Dis. 16, 3540–3542. 10.12102/j.issn.1672-1349.2018.23.044

[B63] Wells G. Shea B. O'Connell J. (2014). The Newcastle-Ottawa Scale (NOS) for Assessing the Quality of Nonrandomised Studies in Meta-Analyses. The Ottawa Hospital Research Institute. Available online at: http://www.ohri.ca/programs/clinical_epidemiology/oxford.asp (accessed April 10, 2022).

[B64] Weng Z. Zhang J. Wang Y. Li L. Xiao Q. Wang Z. . (2002). Clinical efficacy of selegiline added to levodopa/decarboxylase inhibitor in Parkinson's disease. J. Contemp. Neurol. Neurosurg. 2, 281–284. 10.3969/j.issn.1672-6731.2002.05.006

[B65] Ye H. Luo L. X. Li F. Liu Q. G. (2014). Clinical observation of Selegiline in combination with L-dopa on patients with parkinson movement disorders. Chin. Med. Herald. 28, 58–61.

[B66] Yi Z. M. Li X. Y. Wang Y. B. Wang R. L. Ma Q. C. Zhao R. S. . (2022). Evaluating the direct medical cost, drug utilization and expenditure for managing Parkinson's disease: a costing study at a medical center in China. Ann. Transl. Med. 10, 330. 10.21037/atm-22-1014 35433954 PMC9011260

[B67] Zhang W. Lu N. Xie H. Yuan H. Zhao J. (2015). Meta-analysis of selegiline combined with levodopa for Parkinson's disease. Chin. J. Mod. Appl. Pharm. 32, 1498–1502. 10.13748/j.cnki.issn1007-7693.2015.12.021

[B68] Zhao W. W. He X. J. Zhang Z. F. Kong L. S. Su J. J. Xie H. J. (2005). Influence of selegiline on dopaminergic neurons in patients with early Parkinson disease. Chin. J. Clin. Rehabil. 9, 190–192. 10.3321/j.issn:1673-8225.2005.09.02912480442

[B69] Zhao W. W. Zhang Z. F. He X. J. Su J. J. Xie H. J. (2004). Comparison of the efficacy of Pergolide, Selegiline and levodopa-benzylzide in the treatment of Parkinson's disease. Chin. J. New Drugs Clin. Rem. 23, 433–436. 10.3969/j.issn.1007-7669.2004.07.01517630819

[B70] Zhuo C. Zhu X. Jiang R. Ji F. Su Z. Xue R. . (2017). Comparison for efficacy and tolerability among ten drugs for treatment of parkinson's disease: a network meta-analysis. Sci. Rep. 8:45865. 10.1038/srep45865 28374775 PMC5379205

