# Supplementary material for: Efficacy and safety of selegiline for the treatment of Parkinson's disease: A systematic review and meta-analysis
Source: Front Aging Neurosci. 2023 Apr 11;15:1134472. doi: 10.3389/fnagi.2023.1134472 (PMC10126343; doi:10.3389/fnagi.2023.1134472)
Supplement: Supplementary file 1 [file Data_Sheet_1.docx]

Supplementary Material

Efficacy and safety of selegiline for the treatment of Parkinson's disease: a systematic review and meta-analysis

Ke Wang^†^, Ze-Hui Liu^†^, Xin-Ya Li, Yan-Fei Li, Jia-Rui Li, Jiao-Jiao Hui, Jing-Xuan Li, Jun-Wen Zhou^*^, Zhan-Miao Yi^*^

*** Correspondence:**

Zhan-Miao Yi. Email: yzm@bjmu.edu.cn

Jun-Wen Zhou. Email: junwen.zhou@ndph.ox.ac.uk

# Supplementary Table 1 The status of studies reported adverse events

|  | Adverse events (Number of studies) | Studies reported corresponding adverse events in selegiline group |
| --- | --- | --- |
| Neuropsychiatric system disorders | Mild transient sleep disorder (12) | Allain 1993, Larsen et al.1999, Lyytinen 2000, Mizuno 2017, Mizuno 2019, Myllyla 1993, Shoulson 2002, Su 2004, Tao 2019, Wei 2018, Zhao 2004, Zhao 2005 |
|  | Vertigo (9) | Allain 1993, Larsen 1999, Lyytinen 2000, Mizuno 2017, Myllyla 1993, Ruggieri 1986, Shoulson 2002, Su 2004, Tao 2019 |
|  | Headache (6) | Allain 1993, Lyytinen 2000, Mizuno 2017, Myllyla 1993, Ruggieri 1986, Tao 2019 |
|  | Hallucination(vivid dreams) (6) | Larsen 1999, Mizuno 2017, Pålhagen 2006, Shoulson 2002, Wei 2018, Weng 2002 |
|  | Fatigue (4) | Lyytinen 2000, Myllyla 1993, Ruggieri 1986, Shoulson 2002 |
|  | Depression (3) | Larsen 1999, Myllyla 1993, Shoulson 2002 |
|  | Abnormal movement (3) | Lees 1993, Myllyla 1993, Tao 2019 |
|  | Somnolence (3) | Mizuno 2017, Mizuno 2019, Tao 2019 |
|  | Memory loss (3) | Myllyla 1993, Tao 2019, Zhao 2004 |
|  | Dyskinesia (2) | Lees 1993, Tao 2019 |
|  | Syncope (1) | Larsen 1999 |
|  | Fuzzy consciousness (1) | Tao 2019 |
|  | Paresthesias (1) | Ruggieri 1986 |
| Musculoskeletal and connective tissue disorders | Back pain (4) | Larsen 1999, Mizuno 2017, Mizuno 2019, Tao 2019 |
|  | Muscle weakness (2) | Shoulson 1993, Tao 2019 |
|  | Sore limbs (1) | Mizuno 2017 |
|  | Dystonia (1) | Lees 1993 |
| Cardiovascular system disorders | Postural hypotension (6) | Larsen 1999, Lyytinen 2000, Mizuno 2019, Ruggieri 1986, Shoulson 2002, Su 2004 |
|  | Elevated blood pressure (3) | Mizuno 2017, Mizuno 2019, Shoulson 2002 |
|  | Palpitations (3) | Lyytinen 2000, Myllyla 1993, Shoulson 1993 |
|  | Myocardial infarction (2) | Larsen 1999, Shoulson 2002 |
|  | Angina pectoris (2) | Larsen 1999, Shoulson 2002 |
|  | Arrhythmia (1) | Shoulson 2002 |
| Gastrointestinal disorders | Nausea and vomiting (13) | Allain 1993, Larsen 1999, Lyytinen 2000, Mizuno 2017, Mizuno 2019, Pålhagen 1998, Pålhagen 2006, Su 2004, Shoulson 1993, Tao 2019, Wei 2018. Weng 2002, Zhao 2005 |
|  | Dry mouth (5) | Lyytinen 2000, Mizuno 2017, Pålhagen 1998, Tao 2019, Zhao 2004 |
|  | Diarrhea (5) | Larsen 1999, Lyytinen 2000, Mizuno 2017, Pålhagen 1998, Pålhagen 2006 |
|  | Gastrointestinal discomfort (5) | Mizuno 2017, Myllyla 1993, Pålhagen 1998, Ruggieri 1986, Tao 2019 |
|  | Constipation (3) | Mizuno 2017, Mizuno 2019, Zhao 2004 |
|  | Bad taste (2) | Allain 1991, Allain 1993 |
|  | Decreased appetite (2) | Mizuno 2017, Tao 2019 |
|  | Dyspepsia (1) | Larsen 1999 |
| Liver and Gallbladder | Liver diseases (1) | Mizuno 2017 |
| Skin or subcutaneous tissue | Skin reaction and edema (2) | Mizuno 2017, Tao 2019 |


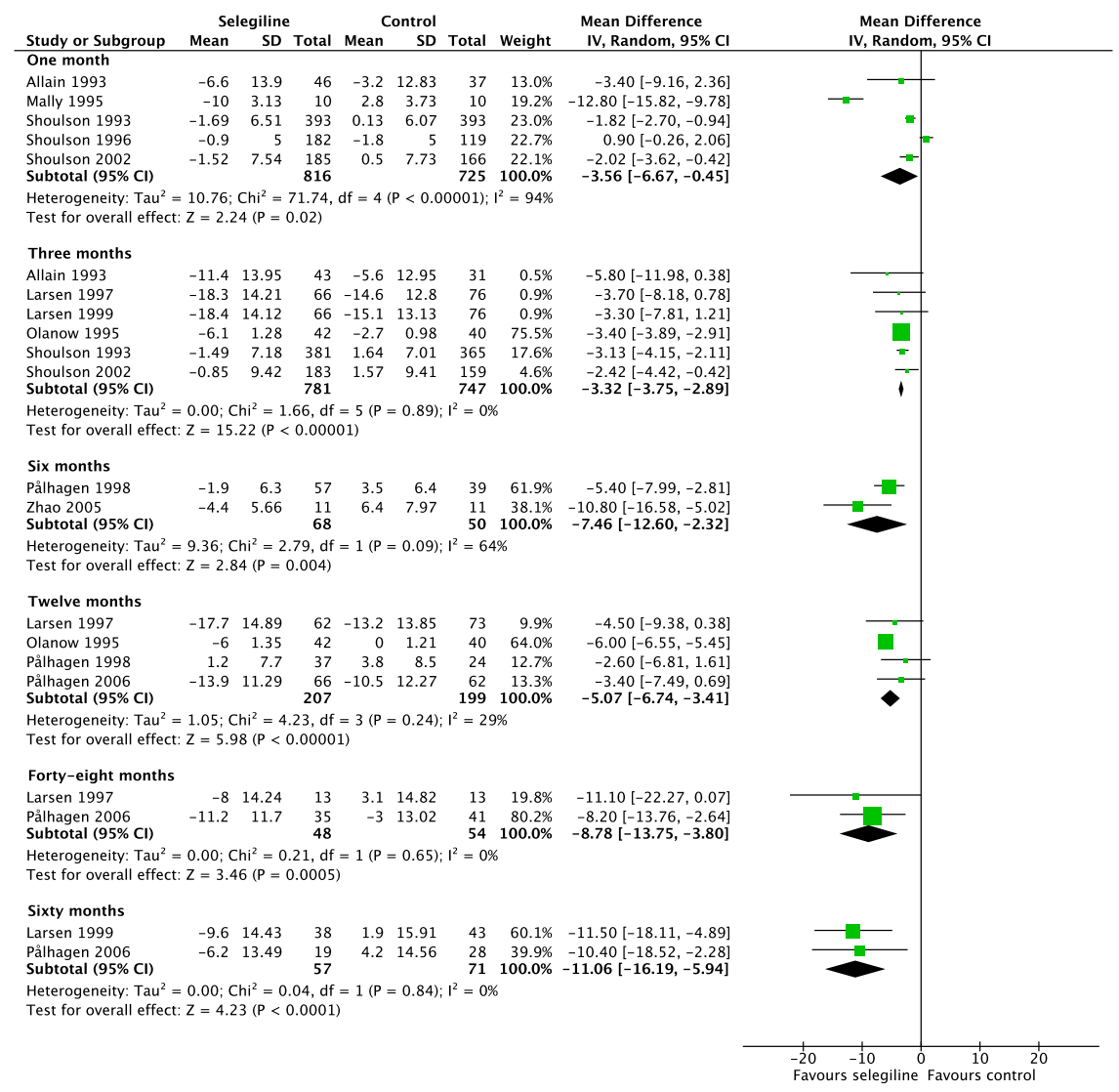


A


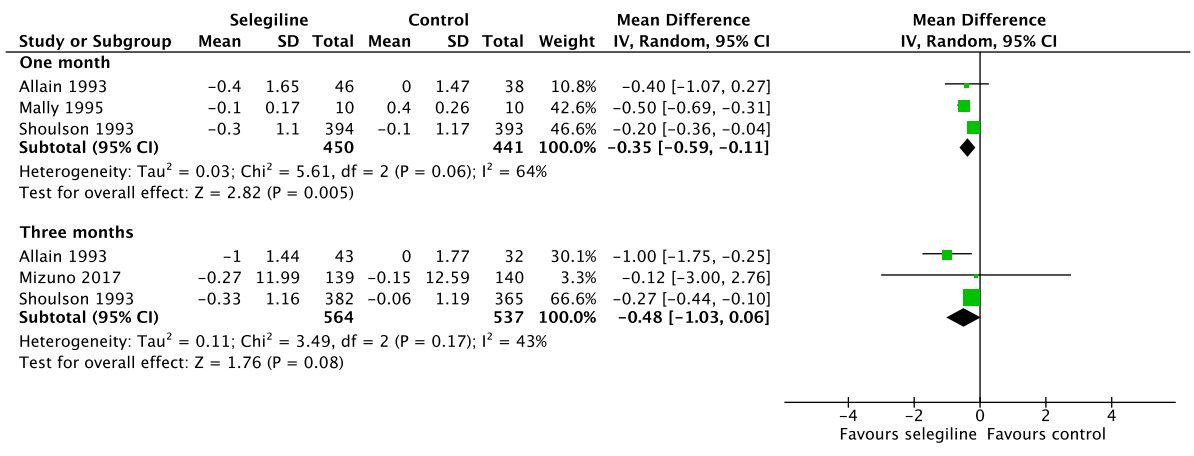


B


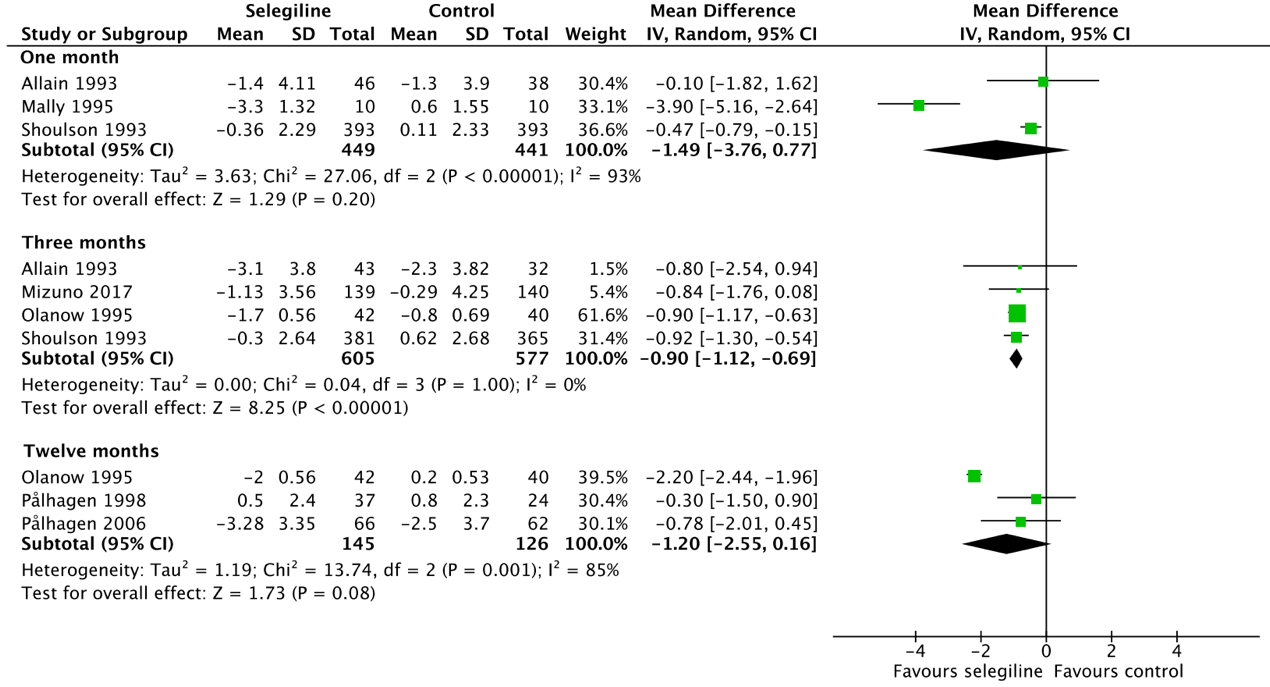


C


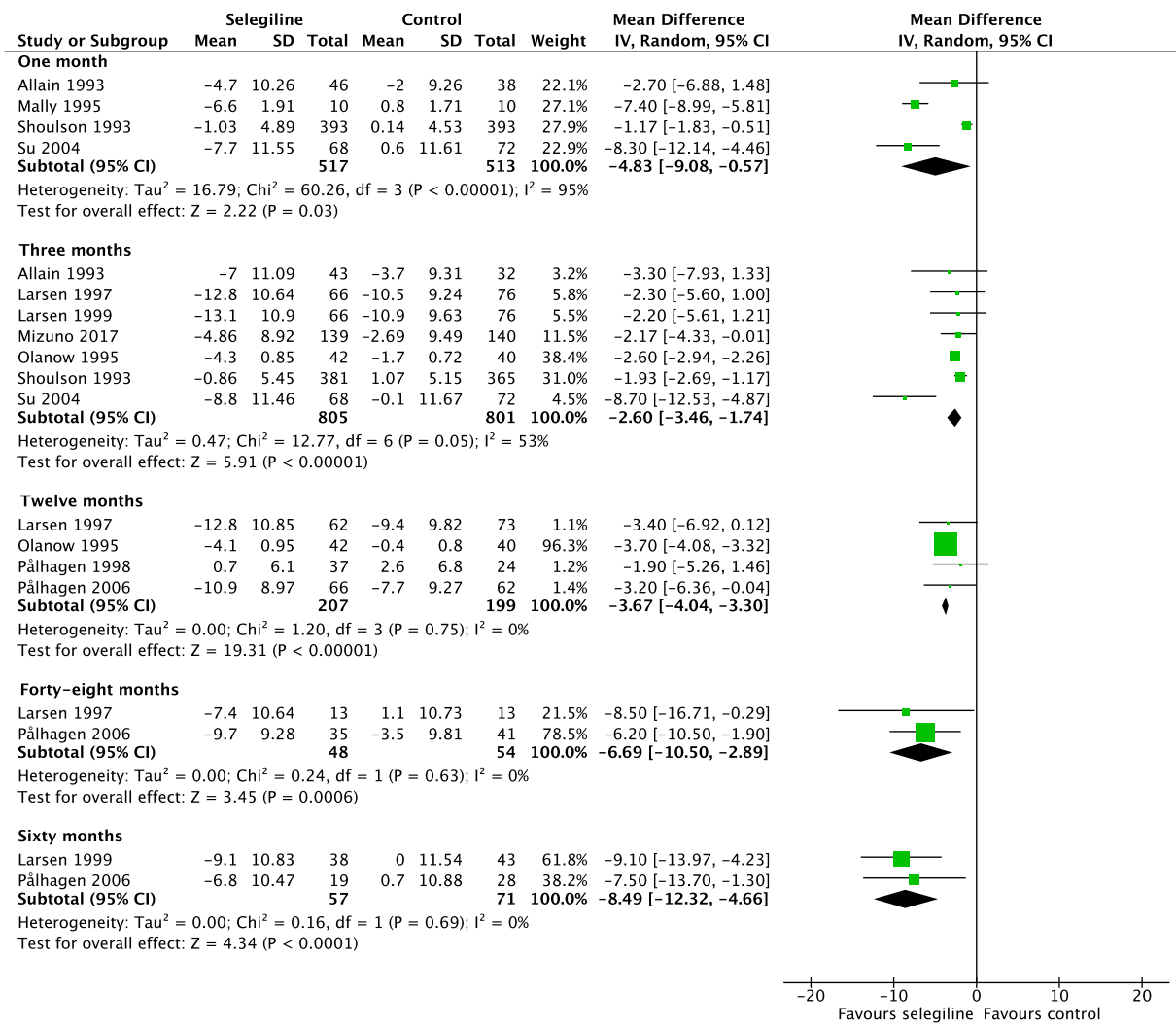


D

**Supplementary Figure 1 Meta-analysis of improvement in total UPDRS (A), UPDRS I (B), UPDRS II (C), UPDRS III (D) score between selegiline and placebo.** UPDRS, Unified Parkinson’s Disease Rating Scale; UPDRS I, mental score; UPDRS II, activities of daily living score; UPDRS III, motor score


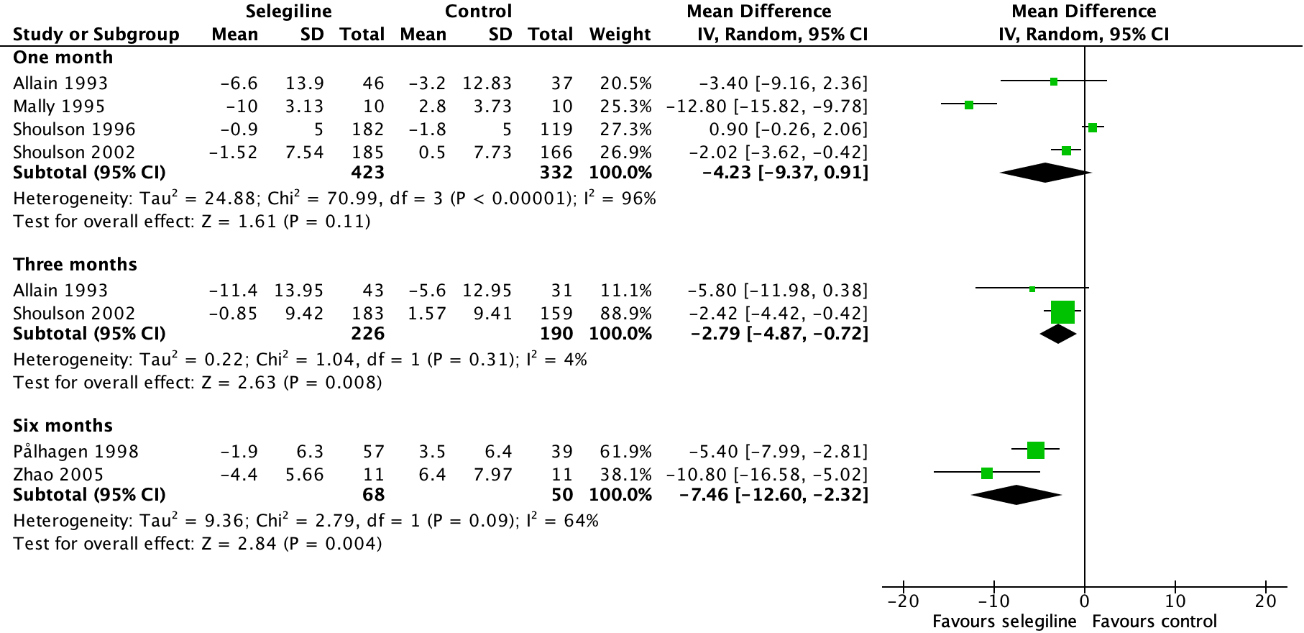


A


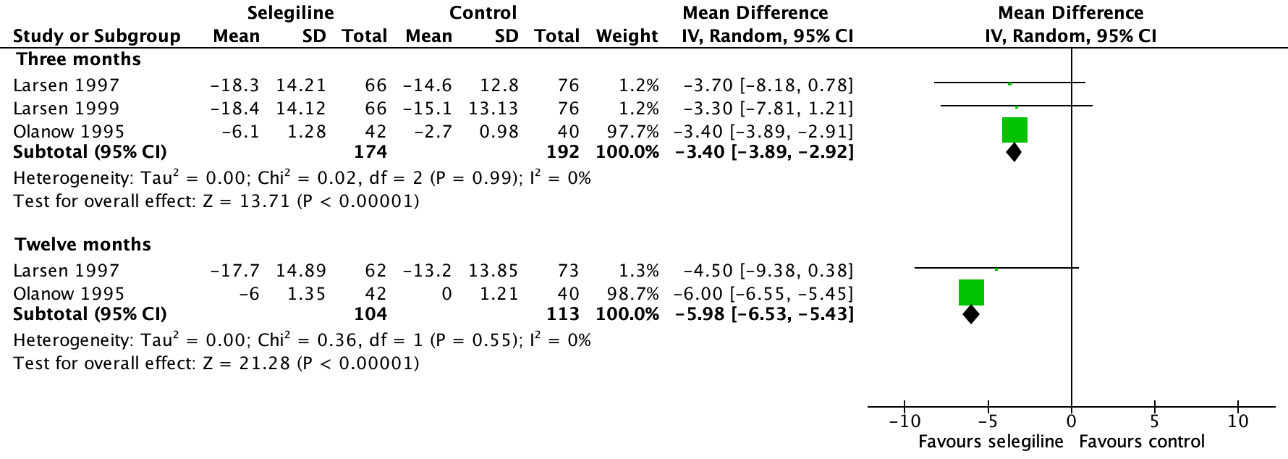


B

**Supplementary Figure 2 Meta-analysis of improvement of total UPDRS score in selegiline monotherapy (A) and in combination with an PD treatment (B), respectively, compared with placebo.** UPDRS, Unified Parkinson’s Disease Rating Scale; PD, Parkinson’s disease


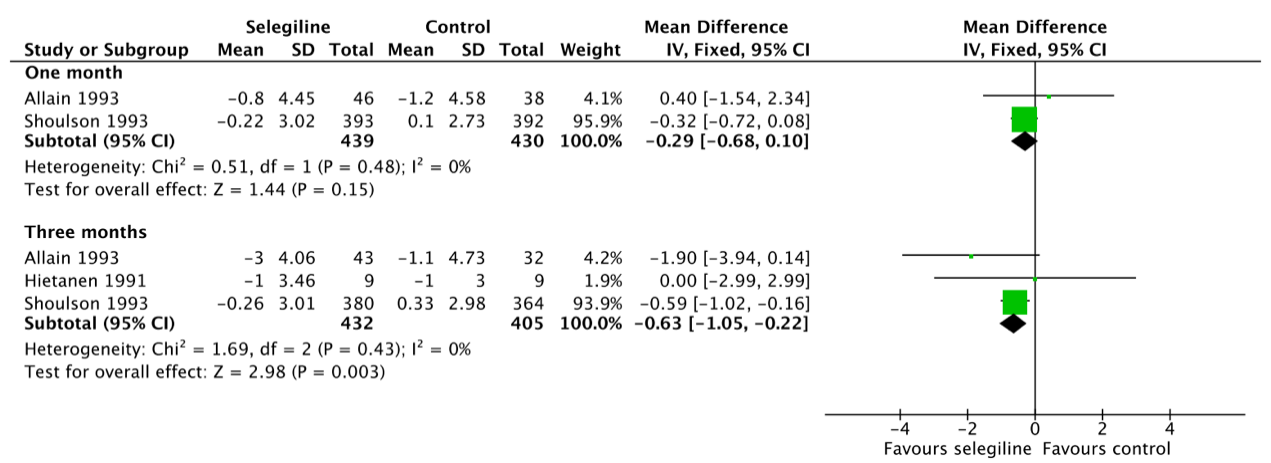


**Supplementary Figure 3 Comparison of change in HAMD score between selegiline and placebo.** HAMD, Hamilton Depression Rating Scale

**
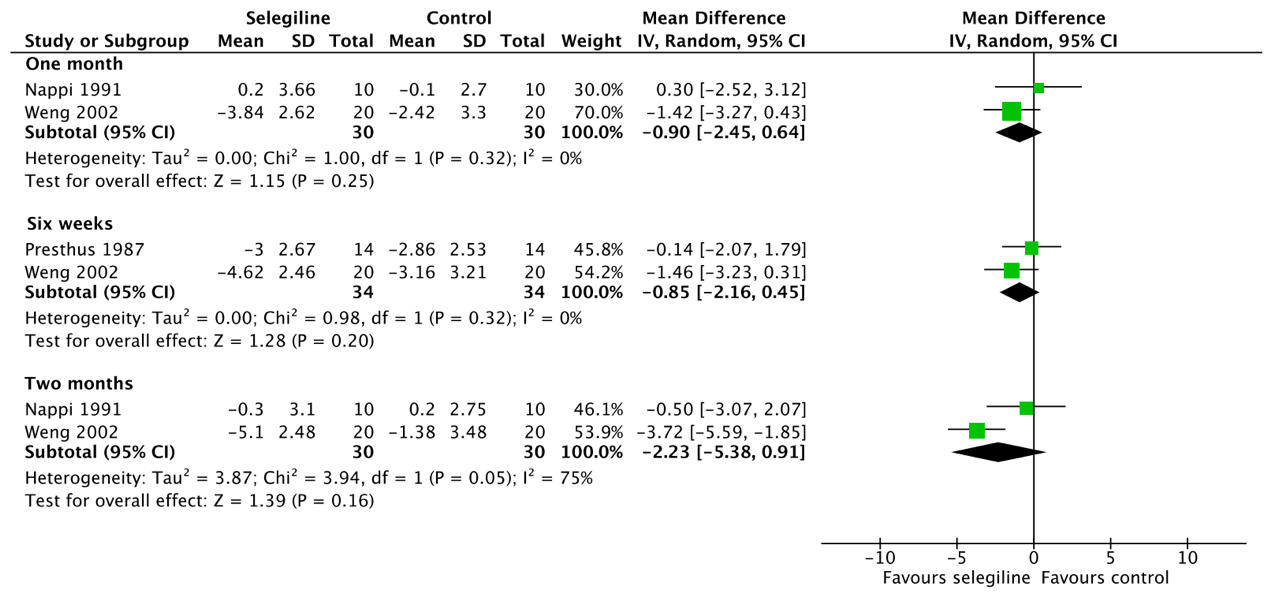
**

**Supplementary Figure 4 Comparison of change in WRS score between selegiline and placebo.** WRS, Webster Rating Scale


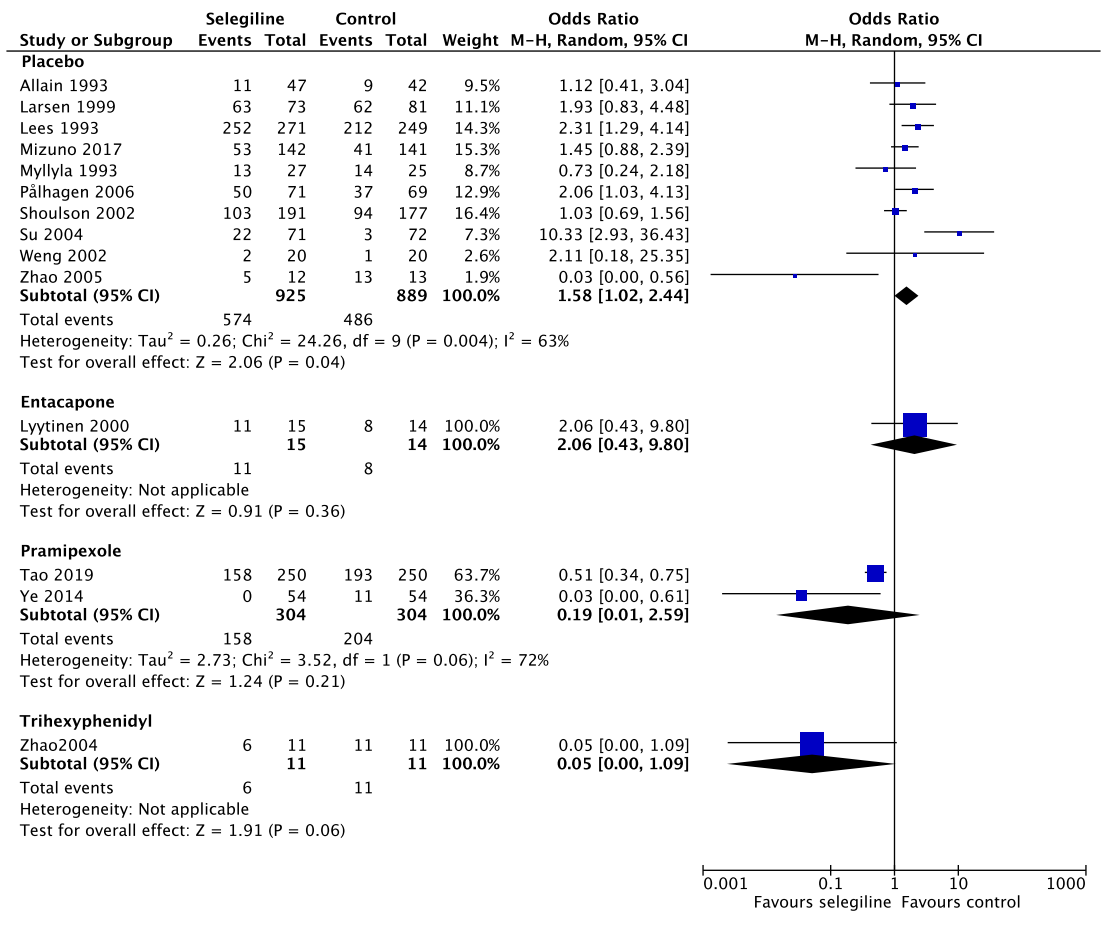


A


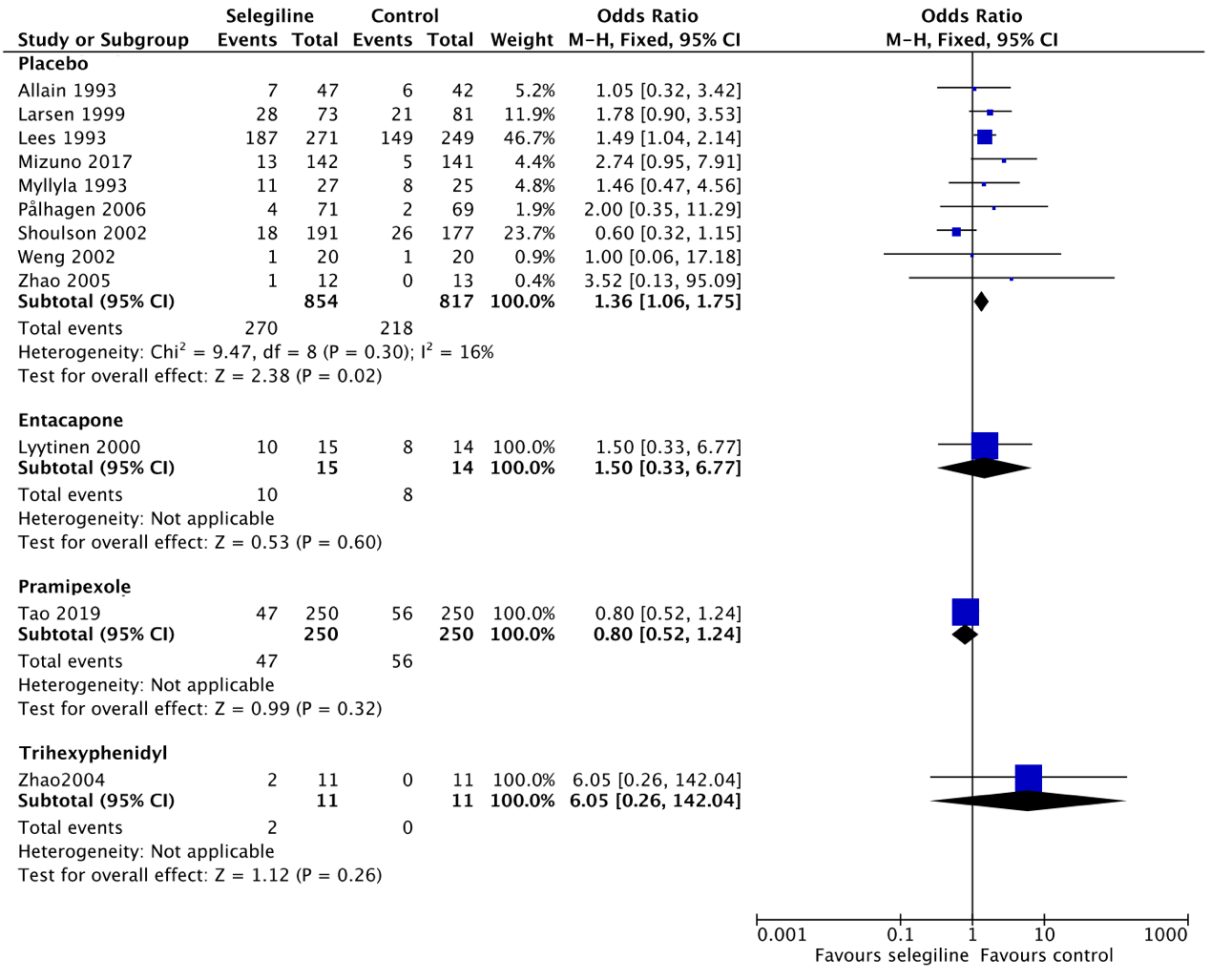


B


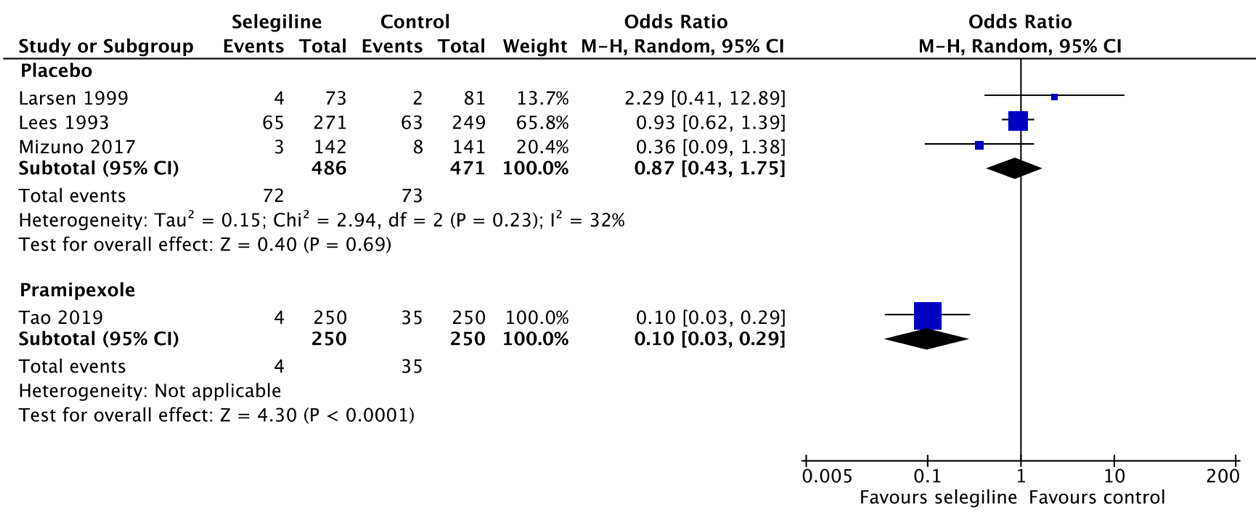


C


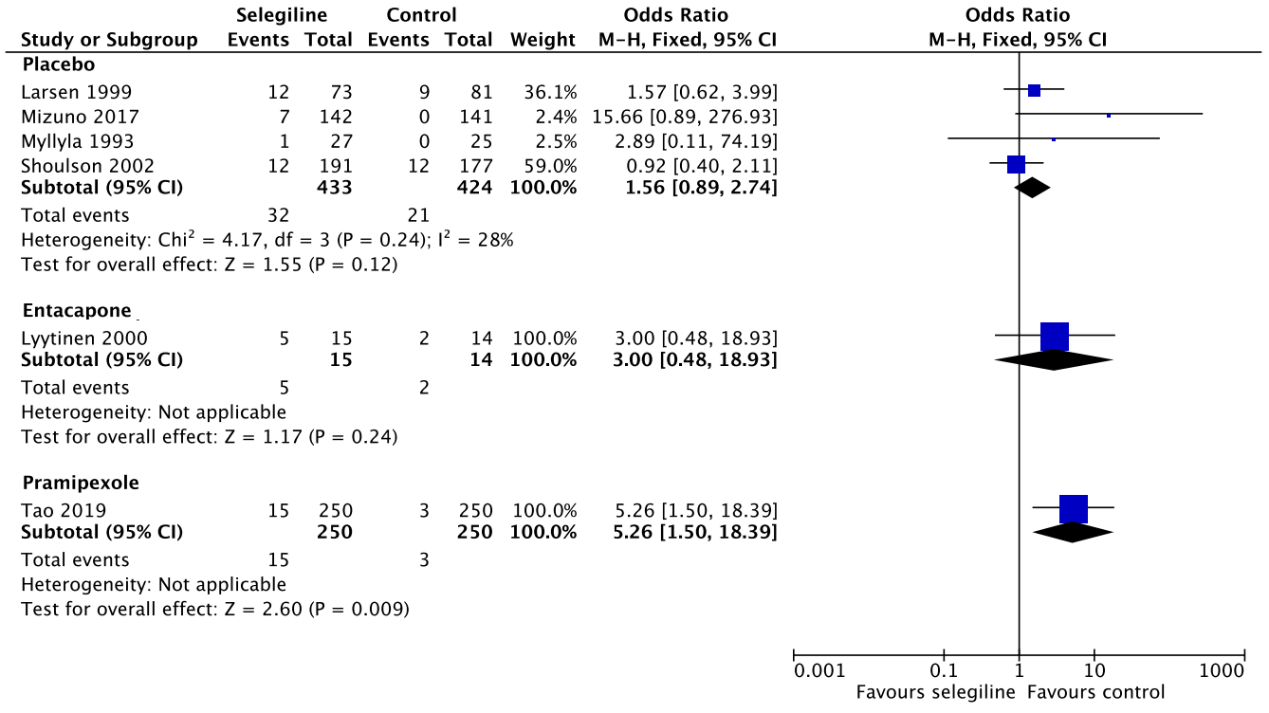


D


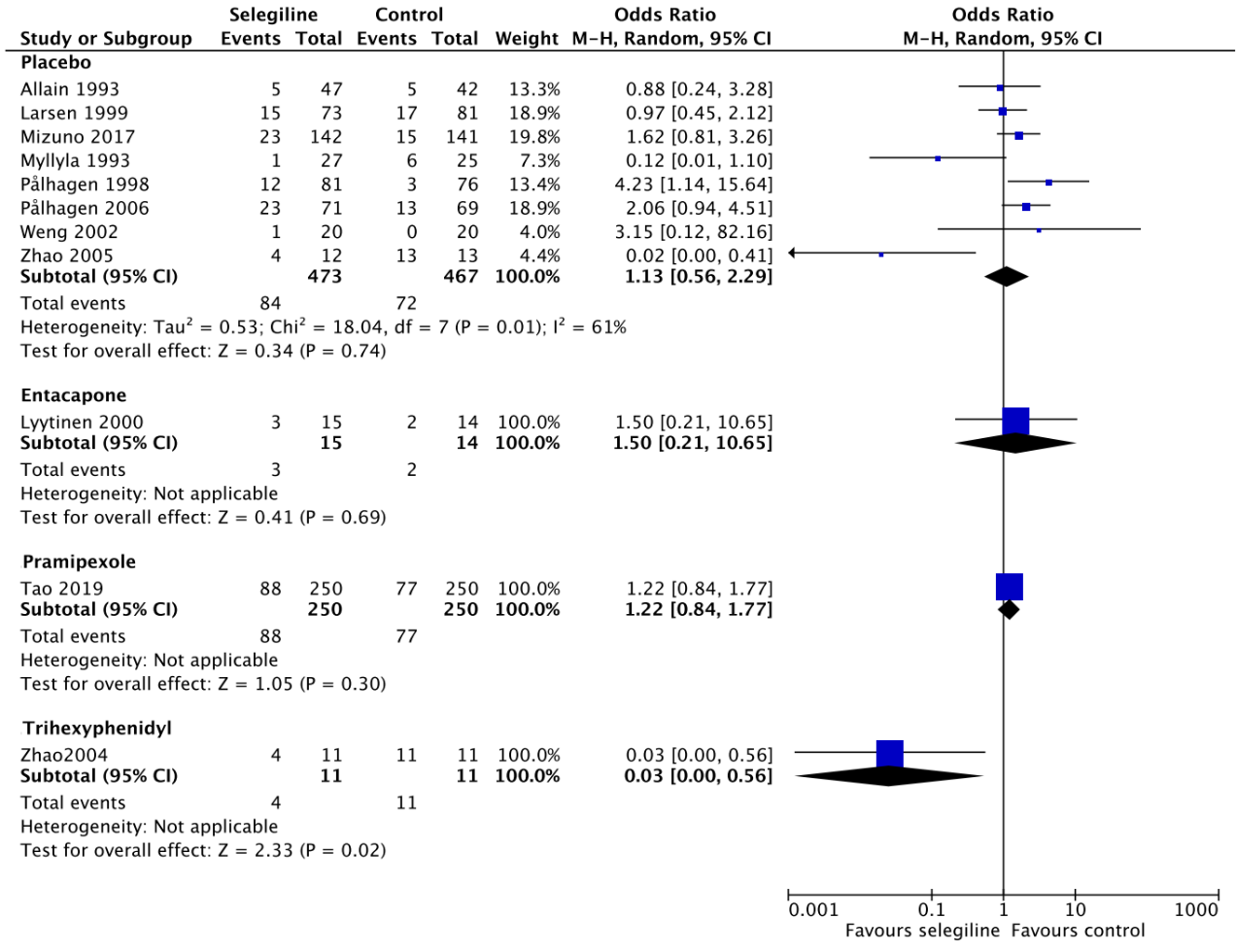


E

**Supplementary Figure 5 Comparison of overall adverse events incidence (A) and classified comparison of neuropsychiatric system disorders (B) , musculoskeletal and connective tissue disorders (C) , cardiovascular system disorders (D) and gastrointestinal disorders (E) incidence between selegiline and control**

**
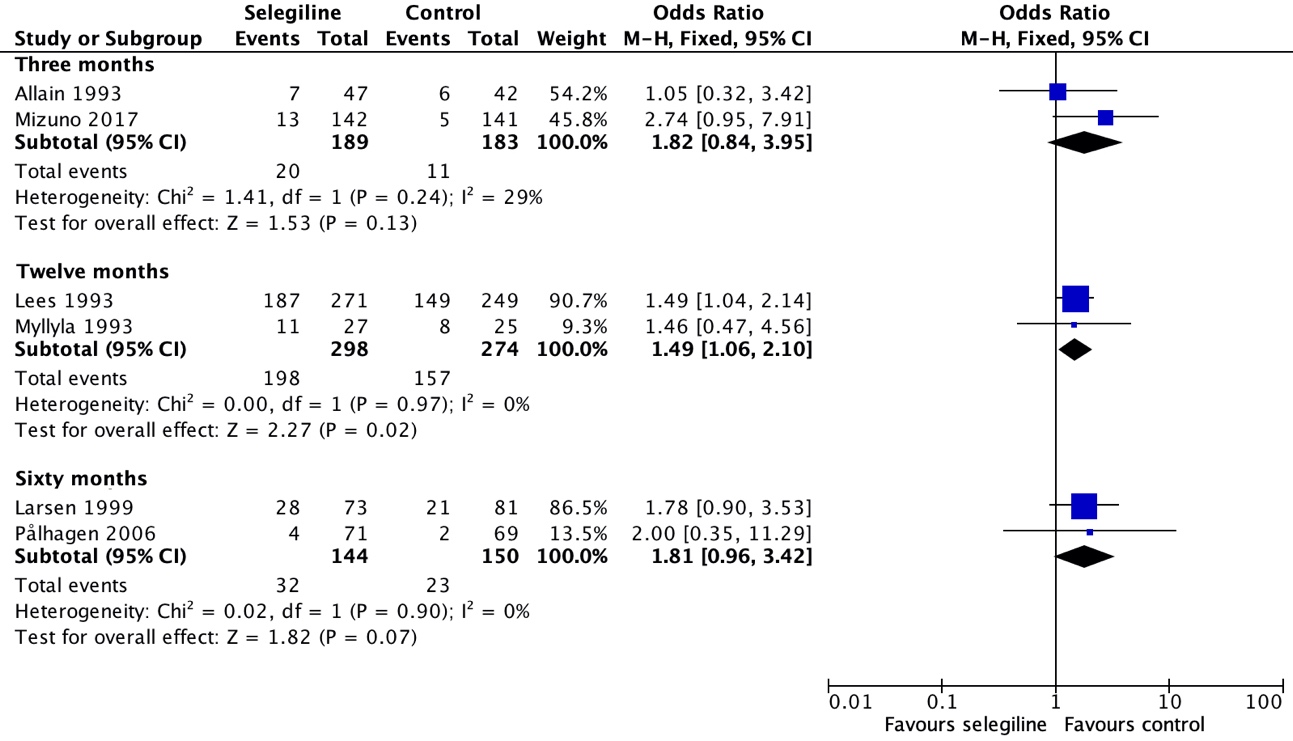
**

**Supplementary Figure 6 Comparison of adverse events in neuropsychiatric system on different treatment durations between selegiline and placebo**

**
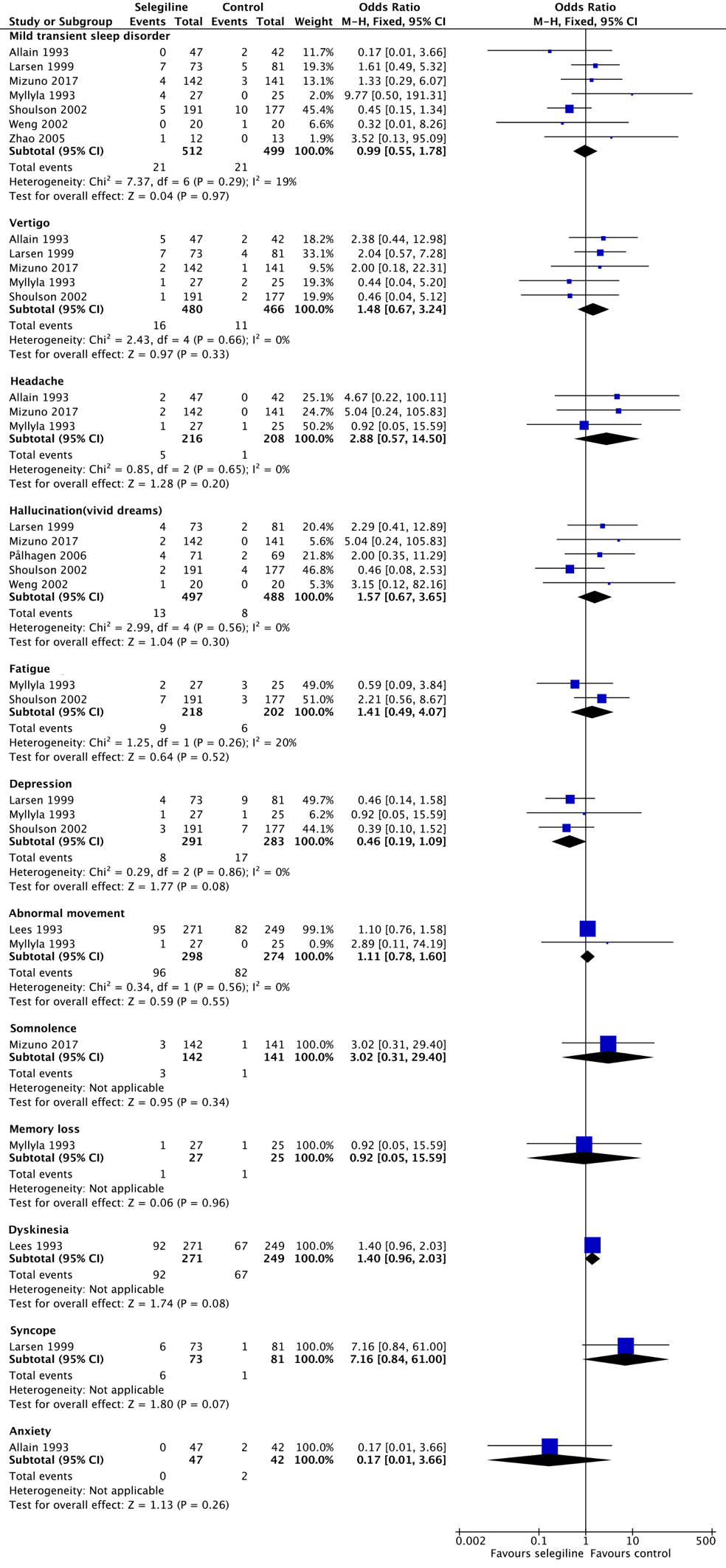
**

**Supplementary Figure 7 Subgroup analysis of adverse events in neuropsychiatric system between selegiline and placebo**
